# Tumor cell death by ferroptosis contributes to an immunosuppressive tumor microenvironment in syngeneic murine models of cancer

**DOI:** 10.1186/s40170-026-00428-3

**Published:** 2026-04-04

**Authors:** Nneka E. Mbah, Damien Sutton, Hanna S. Hong, Rashi Singhal, Rosa E. Menjivar, Matthew D. Perricone, Heather Giza, Peter Sajjakulnukit, Amy L. Myers, Zeribe C. Nwosu, Jonathan M. Alektiar, Jason Lin, Daniel Long, Anthony C. Andren, Li Zhang, Howard C. Crawford, Timothy L. Frankel, Marina Pasca di Magliano, Luigi Franchi, Yatrik M. Shah, Costas A. Lyssiotis

**Affiliations:** 1 Department of Molecular & Integrative Physiology, University of Michigan, Ann Arbor, MI USA; 2 Department of Internal Medicine, Division of Gastroenterology and Hepatology, University of Michigan, Ann Arbor, MI USA; 3 Rogel Cancer Center, University of Michigan, Ann Arbor, MI USA; 4 Graduate Program in Immunology, University of Michigan, Ann Arbor, MI USA; 5 Graduate Program in Cancer Biology, University of Michigan, Ann Arbor, MI USA; 6 Department of Surgery, University of Michigan Medical School, Ann Arbor, MI USA; 7 Department of Pediatrics, University of Michigan Medical School, Ann Arbor, USA; 8 Rogel and Blondy Center for Pancreatic Cancer, Rogel Cancer Center, University of Michigan, Ann Arbor, MI USA

## Abstract

Pancreatic ductal adenocarcinoma (PDAC) is characterized by profound metabolic rewiring and a strongly immunosuppressive tumor microenvironment, both of which contribute to poor therapeutic responses. Immunogenic cell death (ICD) represents a potential strategy to overcome immune suppression by coupling tumor cell death to anti-tumor immune activation. Here, we investigated whether targeting amino acid metabolism in PDAC can induce ICD and promote tumor immunity. Through a focused metabolic screen in a panel of syngeneic mouse cancer cell lines, we identified cysteine restriction as a robust inducer of multiple damage-associated molecular patterns (DAMPs) in vitro, hallmark features of ICD. In addition to driving DAMPs, cystine-deprived tumor cells also promoted dendritic cell phagocytosis, maturation, and proinflammatory cytokine production in vitro. Because cysteine deprivation is a known trigger of ferroptosis, we further demonstrated that pharmacologic inhibition of glutathione peroxidase 4 (GPX4) similarly elicited ICD-associated features, which were reversible by the ferroptosis inhibitor Ferrostatin-1 (Fer-1). To define additional immune-modulatory signals associated with ferroptosis, we performed metabolomic and lipidomic profiling of cells undergoing, but not yet committed to, ferroptotic death. These analyses revealed selective release of immunosuppressive metabolites and oxidized phospholipids. Consistent with this, conditioned media from ferroptotic cells impaired CD8⁺ T cell proliferation and cytotoxicity in vitro. Thus, together our results indicated that the induction of ferroptotic immunogenic cell death led to the release of both pro- and anti-inflammatory signals. Subsequent analysis in vivo revealed that ferroptotic tumor cells predominantly contributed to a tumor-protective environment. In particular, tumors inoculated with ferroptotic cells were enriched with immunosuppressive myeloid cells and exhibited reduced populations of tumor-infiltrating CD8^+^ T cells. Further investigation using immune compromised mice suggested that ferroptotic cells may suppress both adaptive and innate immune responses. Collectively, these results underscore the complex and highly context-dependent effects of ferroptosis on tumor immunity, highlighting the critical importance of in vivo models to determine true immunogenic potential within the tumor microenvironment.

**Supplementary Information:**

The online version contains supplementary material available at 10.1186/s40170-026-00428-3.

## Background

Pancreatic cancer is the third leading cause of cancer mortality in the United States, with an overall 5-year survival rate of 13% [1, 2]. Over 90% of these pancreatic malignancies are pancreatic ductal adenocarcinoma (PDAC) [3]. Incidence of PDAC has been steadily increasing over the past two decades and it is projected to become the second-leading cause of cancer-related death by 2030 [4].

Challenges in treating PDAC result in part from features of the tumor microenvironment (TME) [5, 6]. Within the TME lies an array of stromal cells that deposit a dense extracellular matrix, which leads to high intratumoral pressure and vascular collapse [7]. Consequences of hypovascularity are poor drug penetration, reduced nutrient delivery, and hypoxia [8–10]. Under these nutrient deregulated and oxygen depleted conditions, Kras mutations help to rewire metabolism in PDAC cells [11–13]. This metabolic reshaping involves increased nutrient recycling and scavenging, as well as metabolic crosstalk among cells in the TME [14–20]. Such metabolic adaptations not only support tumor survival but also undermine immune function by depleting nutrients essential for cytotoxic T cells and generating anti-inflammatory metabolites [21, 22]. Immune function in the TME is further impaired by the presence of regulatory T cells, myeloid-derived suppressor cells, and tumor-associated macrophages, which act collectively to secrete factors that impede anti-tumor immunity [23]. Together, these architectural, metabolic, and cellular elements suppress anti-tumor immune responses and promote immune evasion [24].

Recent advances in immune-based therapies are transforming treatment for many cancers by eliciting durable anti-tumor responses [25–28]. These successes highlight the importance of targeting the immune system as a means of eliminating tumors. Immunogenic cell death (ICD) is a specific type of cell death that alerts the immune system by releasing immune stimulatory signals, engaging nearby immune cells. Thus, inducing ICD in cancer cells may offer a therapeutic approach to enhance the anti-tumor immune response and overcome the immunosuppressive TME of PDAC. Indeed, various cancer therapies have been shown to synergize with immune checkpoint inhibitors and other immunomodulatory agents through the induction of immunogenic signals, increasing tumor antigen visibility and relieving the immunosuppressive TME [29–39].

For cell death to be considered immunogenic, dying tumor cells must produce antigens that are recognized by the host immune system and release adjuvants, which strengthen existing immune responses by facilitating the recruitment and activation of immune effectors [40–42]. Adjuvants secreted during ICD are known as damage-associated molecular patterns (DAMPs), which often include externalized calreticulin (ecto-calreticulin), adenosine triphosphate (ATP), and High Mobility Group Box 1 (HMGB1), that bind to pattern recognition receptors on innate immune cells [43, 44]. Although ICD is highly context dependent, and may take the form of immunogenic apoptosis, necroptosis, and pyroptosis, among others [45–47], all forms of ICD trigger the activation of the innate immune system via DAMPs and subsequently prime cytotoxic T lymphocytes through the presentation of tumor antigens [48–51]. In tumors, this process, which coordinates the recruitment and activation of the innate and adaptive immune compartments, allows immunologically “cold” tumors—characterized by poor immune infiltration and activity—to be recognized and targeted by the host immune system [52].

As noted above, metabolic dysregulation is a hallmark of PDAC cells, which meet their biosynthetic demands through rewired metabolism and the activation of various nutrient acquisition programs. While these unique metabolic adaptations promote the survival of PDAC cells in harsh environmental conditions, they also create dependencies that may be exploited therapeutically to induce ICD [53–59]. Targeting these key metabolic pathways in tumor cells to promote cell death while simultaneously facilitating the release of DAMPs that alert immune effectors may help to overcome the suppressive PDAC TME and reinvigorate the anti-tumor immune response. Based on these considerations, we set forth to investigate metabolism-induced ICD.

Using a diverse panel of syngeneic murine cancer cell lines, we first investigated whether perturbing amino acid metabolism can induce features of ICD. By removing individual amino acids from culture media, we found that cysteine restriction causes the release of canonical ICD DAMPs from multiple cancer cell types, including PDAC. We subsequently demonstrated that the presence of ICD features following cysteine deprivation is a consequence of ferroptosis, a metabolic form of cell death marked by dysregulated management of lipid oxidation. While the direct induction of ferroptosis in PDAC cells led to secretion of DAMPs, lipidomic and metabolomic analysis of ferroptotic cells also revealed the selective release of immune-modulating metabolites and lipids. Thus, despite the clear role of ferroptosis in inducing features of ICD in vitro, in vivo vaccination with ferroptotic tumor cells resulted in the promotion of an immunosuppressive TME, evidenced by tumor outgrowth at vaccination sites, infiltration of suppressive myeloid cells, and restriction of cytotoxic T cells. These findings add to the body of evidence highlighting the complex role of ferroptosis in tumor immunity and emphasize the importance of considering cell models and environmental context when studying cell death and immunity [60–63].

## Methods

### Cancer cell lines and cell culture

Murine E.G7-OVA (EG7) T lymphoblasts, which were derived from the C57BL/6 (H-2 b) mouse lymphoma cell line EL4 (ATCC TIB-39), were purchased from the American Type Culture Collection (ATCC). Murine mT3-2D and mT4-2D PDA cell lines were derived from KPC tumors (Pdx1-Cre; LSL-KrasG12D/+; LSL-Trp53 R172H/+) in a C57BL/6J background [64] and were obtained under a material transfer agreement with D. Tuveson (Cold Spring Harbor Laboratory). The KPC-65671 cell line was derived from a KPC mouse tumor [65] in a pure FBVN genetic background, while the iKRAS 9805 cell line (also known as iKras*3 [66]) was derived from a mixed mouse background with the following genotype: ptf1a-cre; TetO-krasG12D; Rosa26rtTa/+; p53R172H/+ [67]. The murine colon carcinoma cell line CT26.CL25 (CT26) was purchased from ATCC and the murine glioma cell line GL261 was a kind gift from M. Castro (University of Michigan). All of these cell lines were cultured in Roswell Park Memorial Institute (RPMI) 1640 (Gibco, 11875093) media supplemented with 10% fetal bovine serum (FBS) (Corning, 35-010-CV) unless otherwise indicated. Murine KPC7940B cells [68] were obtained under a material transfer agreement with G. Beatty (University of Pennsylvania), and murine MC38 [69] and MC38-OVA colon adenocarcinoma cells were a kind gift from M. Green (University of Michigan). These cells were cultured in Dulbecco’s Modified Eagle Medium (DMEM) (Gibco, 11965092) supplemented with 10% FBS. All cells were cultured at 37 °C and 5% CO2 and were routinely screened for mycoplasma using MycoAlert PLUS Mycoplasma Detection Kit (Lonza, LT07-710). All data included in this manuscript were generated with mycoplasma negative cell cultures. The identity of all cell lines was confirmed by STR profiling.

### Amino acid dropout studies

#### Amino acid dropout media

Amino acid dropout assays used standard media with addition of 10% dialyzed FBS (Cytiva, SH30079.03) as controls or an in-house media formulation that identically matched standard media composition and concentration minus the amino acids indicated in each study. RPMI base media was purchased from United States Biological (R9010-02). The following amino acids were purchased from Sigma-Aldrich and were used at the indicated concentrations: 200 mg/L L-arginine (A8094-25G); 50 mg/L L-Asparagine (anhydrous) (A4159-25G); 20 mg/L L-Aspartic Acid (A7219-100G); 65.2 mg/L L-Cystine dihydrochloride (C6727-25G); 300 mg/L L-Glutamine, (G8540-25G); 20 mg/L L-Glutamic Acid (G8415-100G); 10 mg/L Glycine (G8790-100G); 15 mg/L L-Histidine (H6034-25G); 20 mg/L Hydroxy-L-Proline (H5534-10MG); 50 mg/L L-Isoleucine, (I7403-25G); 50 mg/L L-Leucine (L8912-25G); 40 mg/L L-Lysine•HCl (L8662-25G); 15 mg/L L-Methionine (M5308-25G); 15 mg/L L-Phenylalanine (P5482-25G); 20 mg/L L-Proline (P5607-25G); 30 mg/L L-Serine (S4311-25G); 20 mg/L L-Threonine (T8441-25G); 5 mg/L L-Tryptophan (T8941-25G); 28.83 mg/L L-Tyrosine +2Na +2H2O (T1145-25G); 20 mg/L L-Valine (V0513-25G).

#### Amino acid dropout assays

EG7 cells lines were washed and seeded in 96 well plates at 40,000 cells per well in standard RPMI or amino acid dropout media (omission of arginine, cystine, glutamine, leucine, serine (+ glycine) or tryptophan) for 24 h and then assessed for viability, ecto-calreticulin, and extracellular ATP release.

GL261 and CT26 cells were seeded in six-well plates at 150,000 and 120,000 cells, respectively, and allowed to adhere overnight. The following day, GL261 and CT26 cells were washed and their media replaced with either standard RPMI or amino acid dropout media (omission of cystine, glutamine or serine (+ glycine)). After 24 h of incubation, cells were assessed for viability, ecto-calreticulin, and extracellular ATP release.

#### Viability and detection of ecto-calreticulin

Cells were washed once in ice-cold Annexin Binding Buffer (Invitrogen, V13246) then resuspended in ice-cold staining solutions containing Annexin V buffer plus Calreticulin-FITC (Novus Biologicals, NBP1-47518F) at a 1:100 dilution or Annexin V-Pacific Blue (Molecular Probes, A35122) at a 1:300 dilution. After 40 min of incubation, staining solutions were removed and cells were washed in ice cold Annexin Binding Buffer. Next, cells were resuspended in ice-cold Annexin V binding buffer containing a 7-AAD viability staining solution (Invitrogen, 00699350), followed by flow cytometry analysis (Bio-Rad Ze5 Cell Sorter).

#### Extracellular ATP

Cells were plated and treated according to assay-specific conditions. At endpoint, extracellular ATP was measured using ENLITEN ATP Assay System Bioluminescence Detection Kit (Promega, FF2000) according to the manufacturer’s protocol.

#### Isolation of BMDCs

BMDCs were derived from C57BL/6J mice purchased from Jackson Laboratory. Cells were flushed with PBS from the isolated femurs and tibias of 6–8 week-old mice and cultured in RPMI 1640 media (Gibco, 11875093), 10% FBS (Corning, 35-010-CV), penicillin-streptomycin 100 U/mL (Gibco, 15140122), 2 mM L-glutamine (Gibco, 25030081), 50 µM 2-mercaptoethanol (Life Technologies, 31350010), 1X non-essential amino acids (Gibco, 11140076), 1 mM sodium pyruvate (Gibco, 11360070), and 20 ng/mL GM-CSF (Abcam, ab9742) for 7 days, with fresh media supplemented every two days. Cultured cells were then collected and used for experiments. BMDCs are identified as floating and loosely attached cells on the dish following culture of bone marrow cells.

#### BMDC phagocytosis and maturation assays

Prior to coculture for phagocytosis assays, EG7 cells (500,000 cells per well in 24-well plates) were stained with 1µM Cell Tracker Green CMFDA Dye (Invitrogen, C2925) in their respective phenol-free, serum-free RPMI 1640 media for 48 h. For coculture, BMDCs were plated in BMDC media at 200,000 cells per well in 6-well plates. EG7 cells were then assayed for viability by trypan blue staining (Gibco, 1525006) and were cultured with BMDCs for 24 h before harvest. BMDCs assessed in phagocytosis assays were stained for 35 min in cold PBS with APC-CD11c (BD Biosciences, 55026). After one wash, BMDCs were resuspended in 1µM Sytox blue (ThermoFisher, S11348). Analysis was performed using FlowJo (v.10.0.8) software. BMDCs were sorted using a Bio-Rad Ze5 Cell Sorter. Single cells were obtained by gating on FSC-A versus FSC-H, followed by SSC-A versus SSC-H. Dead cells were excluded by SYTOX Blue. BMDCs that successfully phagocytized target cells were identified as CD11c+CMFDA+.

For BMDC maturation assays, EG7 cells (500,000 cells per well in a 24-well plate) were cultured in their assay-specific media for 48 h. BMDCs were plated in BMDC media at 200,000 cells per well in 6-well plates. Target cells were assayed for viability and then were added at indicated ratios with BMDCs for 24 h before harvest. BMDCs assessed in maturation assays were stained for 35 min in cold PBS with APC-CD11c (BD Biosciences, 550261), PeCy7-CD86 (BD Biosciences, 560582), PE-Cy7-anti-CD80 (eBioscience, 25080182), and FITC-MHCII (eBioscience,11532181). After one wash, BMDCs were resuspended in 1µM Sytox blue (Invitrogen, S11348). Analysis was performed using FlowJo (v.10.0.8) software. BMDCs were sorted using a Bio-Rad Ze5 Cell Sorter. Single cells were obtained by gating on FSC-A versus FSC-H, followed by SSC-A versus SSC-H. Dead cells were excluded by SYTOX Blue. Mature BMDCs cells were identified as a percent of CD11c^+^ CD86^+^ MHCII^+^ and CD11c^+^ CD80^+^ MHCII^+^.

### EG7 cell assays

#### Cystine viability assay

Cystine dropout media was formulated in-house using RPMI 1640 Medium Modified (Sigma, R7513-100ML) which was supplemented with 15 mg/L L-Methionine, 300 mg/L L-Glutamine, and 65.2 mg/L, 6.52 mg/L, 0.652 mg/L, 0.0652 mg/L, or 0.00652 mg/mL L-Cystine. All media was supplemented with 10% FBS and was prepared fresh for each assay. Cells were washed and seeded in 96-well plates at 40,000 cells per well in 200 µL of each media condition with or without 2 µM Fer-1 for 96 h. At end point, cell viability was measured using CellTiter-Glo^®^ 2.0 Cell Viability Assay.

#### RSL3 and erastin viability, ecto-calreticulin expression, and extracellular ATP assays

For viability and ecto-calreticulin measurements, cells were washed and seeded in 96-well plates at 40,000 cells per well in 200 µL media containing each indicated drug condition with or without 2 µM Fer-1 for 24 h. At end point, cells were centrifuged and 100 µL of media was taken for extracellular ATP measurements. Cells were then processed for flow cytometry to measure ecto-calreticulin or cell viability.

#### Lipid peroxidation assay

Cystine-free media was formulated in house using RPMI 1640 Medium Modified (Sigma, R7513-100ML) supplemented with 15 mg/L L-Methionine and 300 mg/L L-Glutamine. Cystine-replete media added 65.2 mg/L L-Cystine (Sigma-Aldrich, 57579-5ML-F) to this formulated media. Cystine-replete media was used for the 3 µM Erastin (Cayman, 17754) and RSL3 (Cayman, 19288) treatment groups. All media was supplemented with 10% FBS. Cells were washed and seeded in 96-well plates at 40,000 cells per well in 200 µL of each media condition with or without 2 µM Fer-1 (Cayman, 17729) for 12 h. At end point, cells were processed for flow cytometry to measure C-11 BODIPY.

#### Lipid ROS measured by C-11 BODIPY

Cells were stained in prewarmed phenol red-free media with low FBS (0.2% FBS) using 2 µM C-11 BODIPY (Invitrogen, D3861) for 30 min at 37 °C and 5% CO_2_. Cells were then washed in ice-cold PBS (Gibco, 10010023) twice before detachment via 0.25% Trypsin-EDTA (Gibco, 25200056). After neutralization and removal of trypsin, staining with 1 µM Sytox Blue (ThermoFisher, S34857) was performed. Cells were promptly analyzed using a Bio-Rad Ze5 Cell Sorter.

#### Generation of GPX4 knockout cells

GPX4 CRISPR–Cas9 constructs were generated using the expression vector pSpCas9(BB)-2A-Puro (PX459) V2.0, which was obtained from Addgene (Plasmid #62988). As described formerly [70], the restriction enzyme BbsI was used to cut the plasmid followed by ligation of mouse GPX4 sgRNA sequences. Oligonucleotide pairs were obtained from the Human GeCKO Library (v2, 3/9/2015). The day prior to transfection, KPC7940B cells were seeded at 200,000 cells per well in a 6-well plate. Lipofectamine^®^ LTX Reagent (Invitrogen, 15338100) in conjunction with PLUS™ (Invitrogen, 11514015) was used to transfect cells with 2.5 µg of pSpCas9-GPX4 according to the manufacturer’s instructions. Selection with 2 µg/mL puromycin began 24 h later. Selection was continued with replacement of 2 µg/mL puromycin media every two days until death of all non-transfected cells. Single cell clones were selected and expanded from successfully transfected cell lines. At the time of transfection and during all subsequent culture of GPX4 KO cells, media was supplemented with 2 µM Fer-1.

### KPC7940B cell assays

#### Time course of extracellular ATP and CXCL1 detection

KPC7940B wild type and GPX4 knockout cells were seeded at 900,000 cells per 10 cm plate in 10 mL DMEM (Gibco, 11965092) supplemented with 10% FBS (Corning, 35-010-CV) for 18 h. Cells were then washed, and media was replaced with 6 mL media containing each indicated drug treatment. Supernatants from each plate were taken at 0, 1-, 2-, 4- and 8-hour time points, centrifuged at 300 g for 5 min at 4 °C and then promptly divided for measuring either extracellular ATP using the ENLITEN ATP Assay System Bioluminescence Detection Kit (Promega, FF2000) or CXCL1 by ELISA through the University of Michigan Immune Monitoring Shared Resource according to the manufacturer’s protocols.

#### Analysis of ecto-calreticulin and lipid peroxidation

KPC7940B wild type and GPX4 knockout cell lines were seeded at 200,000 cells per well in a 6-well plate for 24 h. Cells were then washed twice with 3 mL PBS, treated with 3 mL of their indicated media for 4 h, and then collected and assessed for ecto-calreticulin and lipid peroxidation by flow cytometry.

#### Analysis of viability

KPC7940B wild type and GPX4 knockout cell lines were washed and seeded at 20,000 cells per 96-well in 200 µL of their indicated media with or without 2 µM Fer-1 for 72 h. At end point, cell viability was measured using CellTiter-Glo^®^ 2.0 Cell Viability Assay.

#### Cell death inhibitor assays

Cell death inhibitors that were tested in this assay included Fer-1 (Cayman, 17729), Bafilomycin A1 (Cayman, 11038), Necrosulfonamide (Cayman, 20844), Z-VAD(Ome)-FMK (Cayman,14463), Necrostatin-1 (Cayman, 11658) and Mitoxantrone (Cayman, 14842). KPC7940B wild type cells were washed and seeded at 40,000 cells per 96-well in 200 µL of media. Cells were co-treated with a cell death inhibitor and either 5 µM imidazole ketone erastin (IKE) (Cayman, 27088), 1 µM RSL3, or DMSO vehicle for 48 h. Cell viability was measured using CellTiter-Glo^®^ 2.0 Cell Viability Assay.

#### Colony forming assay

KPC7940B wild type and GPX4 knockout cell lines were washed and seeded at 250 cells per 6-well in 3 mL of media with or without 2 µM Fer-1 for 6 days. Media was then removed, and cells were washed in ice-cold PBS and were fixed with 100% methanol for 10 min. After removal of methanol, cells were stained with 0.5% crystal violet solution for 15 min. Crystal violet was then aspirated, followed by repeated washing steps to remove the remaining crystal violet.

#### Dose response curves

KPC7940B cells were washed and seeded in 96-well plates at 40,000 cells per well in 200 µL of media with varying concentration of IKE, RSL3, or cystine, each with or without 2 µM Fer-1 for 48 h. Cell viability was then measured using CellTiter-Glo^®^ 2.0 Cell Viability Assay. Cystine dropout media was formulated in-house using RPMI 1640 Medium Modified (Sigma, R7513-100ML) supplemented with 15 mg/L L-Methionine and 300 mg/L L-Glutamine. L-Cystine (Sigma-Aldrich, 57579-5ML-F) was added back at various concentrations, with 100% cystine equal to 65.2 mg/L. All media was also supplemented with 10% FBS. IC_50_ values were calculated using Prism analyzation tools.

### Metabolomic profiling

#### Metabolomics sample preparation

For both media supernatant and cell pellet metabolomic analysis, PDAC cells were plated in triplicate in 10 cm plates at a density of 900,000 cells in DMEM supplemented with 10% FBS. Two additional plates were prepared in parallel for protein quantification and subsequent normalization of samples, as well as for flow cytometry to measure viability by 7-AAD. After seeding overnight, the culture medium was removed, cells were washed with PBS, and fresh medium was added containing the indicated conditions. Cells were then incubated for 4 h, at which point supernatants and pellets were collected for metabolite extraction. To collect metabolites from media supernatants, 1.5 mL of culture medium was taken from each plate, transferred to a tube, and spun at 300 g for 5 min at 4 °C. Next, 200 µL of supernatant was transferred to a new tube, followed by the addition of 800 µL of ice-cold methanol. For metabolite extraction from cell pellets, the remaining medium was aspirated, and the cells were washed once with 4 mL PBS. Next, 4 mL of ice-cold methanol/water 1:4 (v/v) was added, and the plate was incubated on dry ice for 10 min. The resulting cell lysates were then transferred into tubes for centrifugation at 12,000 g. For each experimental group, the volume of supernatant collected from cell lysates for drying was adjusted according to the protein content measured from the cell pellets of the parallel plate. Samples were dried using a SpeedVac Vacuum Concentrator (model: SPD1030).

#### Targeted metabolomics

The dried supernatants were reconstituted in methanol diluted in water (1:1) and analyzed by LC-MS according to a previously described protocol [71]. Data processing was performed using Agilent MassHunter Workstation Quantitative Analysis for QQQ software (version 10.1, build 10.1.733.0). Heatmaps were generated using MetaboAnalyst 6.0 [72].

Raw data are provided as Supplementary File 1.

### Lipidomic profiling

#### Lipidomics sample preparation

For both media supernatant and cell pellet lipidomic analysis, PDAC cells were plated in triplicate in 15 cm plates at a density of 10,000,000 cells in DMEM supplemented with 10% FBS. An additional plate was prepared in parallel for protein quantification and subsequent normalization of samples. After incubating overnight, the culture medium was removed, cells were washed with PBS, and fresh medium was added containing the indicated conditions. Cells were then incubated for four hours, at which point supernatants and pellets were collected for lipid extraction. To collect lipids from media supernatants, 1.5 mL of culture medium was taken from each plate, transferred to a tube, and centrifuged at 300 g for 5 min at 4 °C. After centrifugation, 1 mL of supernatant from each replicate were immediately transferred to a new tube and frozen at -80 °C along with 1 mL of cell-free culture media as a control. To collect lipids from cell pellets, the remaining medium was aspirated, and the cells were washed twice with 15 mL PBS. Cells were collected from plates using trypsin, followed by centrifugation at 300 g for 5 min at 4 °C. Trypsin was then removed and cell pellets were washed twice in 15 mL PBS. After aspirating the second wash, cells pellets were resuspended in 1 mL PBS containing 200 µM diethylenetriaminepentaacetic acid (Sigma, D6510-10G). Next, one part methanol was added, after which samples were promptly frozen at -80 °C. Protein content was measured using the cell pellets of the parallel plate. These protein measurements were used for normalizing lipids from cell pellets. Samples were transported to Cayman Chemical on dry ice.

#### Targeted and untargeted lipidomics

Lipidomics analysis was performed as detailed below by Cayman Chemical Company, Ann Arbor, Michigan. Upon receipt, samples were stored at -80 °C until the time of analysis. After thawing, lipids were extracted using a methyl-tert‐butyl ether (MTBE)‐based liquid‐liquid extraction method. Samples were thawed on ice in the original tubes before adding 500 µL PBS/methanol 1:1 (v/v) and then 100 µL methanol containing 50 ng each of the following internal standards for targeted lipidomics: PC(15:0/18:1-d7), PC(15:0/18:1-d7), PE(15:0/18:1-d7), PE(15:0/18:1-d7), PG(15:0/18:1-d7), PG(15:0/18:1-d7), PI(15:0/18:1-d7), PI(15:0/18:1-d7), PS(15:0/18:1-d7), PS(15:0/18:1-d7), PC(15:0/18:1-d7), PE(15:0/18:1-d7), PG(15:0/18:1-d7), PI(15:0/18:1-d7), and PS(15:0/18:1-d7). For untargeted metabolomics, 500 µL PBS/methanol 1:1 (v/v) and then 100 µL methanol containing 50 ng each of the following internal standards was added: TG(15:0/18:1-d7/15:0), DG(15:0/18:1-d7/0:0), Cer(d18:1-d7/15:0), SM(d18:1/18:1-d9), PG(15:0/18:1-d7), PC(15:0/18:1-d7), PI(15:0/18:1-d7), PS(15:0/18:1-d7), LysoPC(18:1-d7), LysoPE(18:1-d7), PE (15:0/18:1-d7). Samples were then transferred into 8‐mL screw‐cap tubes, and then 1.125 mL methanol and 5 mL MTBE were added. After vigorous mixing, samples were incubated at room temperature on a tabletop shaker for 45 min. For phase separation, 1.25 mL water was added, and samples were vortexed and centrifuged for 15 min at 2000 x g. The upper organic phase of each sample was carefully removed using a Pasteur pipette, transferred into an empty glass round‐bottom tube, and dried under vacuum in a SpeedVac concentrator. The dried lipid extracts were resuspended in 200 µL HPLC mobile phase A/mobile phase B 3:1 (v/v) for targeted LC-MS/MS analysis. For untargeted LC-MS/MS analysis, resuspended extracts were dried again under vacuum in a SpeedVac concentrator, then resuspended in 100 µL 1‐butanol/methanol 1:1 (v/v). For targeted LC-MS/MS, extracted lipid samples (20 µL) were injected onto a Solex ExionLC Integrated System coupled to a Sciex QTrap 6500 + mass spectrometer. Lipid separation was performed using a reverse-phase Kinetex HILIC core shell column (2.6 μm, 150 × 2.1 mm, Phenomenex) maintained at room temperature, with a flow rate of 200 µL/min. The mobile phases consisted of hexane/isopropanol (30:40, v/v) (Mobile Phase A) and hexane/isopropanol/water (30:40:7.5, v/v/v) with ammonium acetate (Mobile Phase B). The chromatographic gradient was as follows: 0 min, 25% B; 1 min, 25% B; 4 min, 60% B; 7 min, 85% B; 10 min, 95% B; 11 min, 25% B; 15 min, 25% B for re-equilibration. Mass spectrometric analysis was performed using electrospray ionization (ESI) in both positive and negative ion modes, with polarity switching. The electrospray voltage was set to 4500 V for both positive and negative modes, with a source temperature of 450 °C. Ion source gas 1 and gas 2 flow rates were set to 35 psi each, and the curtain gas was set to 35 psi. The collision gas was set to low, with collision energy set to -30 eV (negative mode) or + 45 eV (positive mode). The declustering potential was set to ± 100 V, entrance potential to ± 10 V, and collision cell exit potential to -7.5 V (negative mode) or + 10 V (positive mode). Data were acquired in MRM (Multiple Reaction Monitoring) mode with a dwell time of 25 ms. For untargeted LC-MS/MS, extracted lipid samples (5 µL) were injected onto an Ultimate 3000 UPLC system (Thermo Scientific) coupled to a Q-Exactive Plus Orbitrap mass spectrometer (Thermo Scientific). Lipid separation was performed using a reverse-phase Accucore C30 column (2.6 μm, 150 × 2.1 mm, Thermo Scientific) maintained at 40 °C, with a flow rate of 300 µL/min. The mobile phases consisted of acetonitrile/water/formic acid (60:40:0.1, v/v/v) with 10 mM ammonium formate (Mobile Phase A) and acetonitrile/isopropanol/formic acid (10:90:0.1, v/v/v) with 10 mM ammonium formate (Mobile Phase B). The chromatographic gradient was as follows: 0 min, 30% B; 5 min, 43% B; 5.1 min, 50% B; 14 min, 70% B; 14.1 min, 70% B; 21 min, 90% B; 28 min, 98% B; 33 min, 30% B for re-equilibration. Mass spectrometric analysis was performed using electrospray ionization (ESI) in both positive and negative ion modes, with polarity switching. The spray voltage was set to 3.0 kV for positive mode and 3.2 kV for negative mode, with a capillary temperature of 350 °C. Sheath gas and auxiliary gas flow rates were set to 60 and 20 (arbitrary units), respectively, and the S-lens RF level was 45. The collision energy was set to 28 eV. Data were acquired in both full MS and data-dependent MS² (dd-MS²) modes, with a mass resolution of 70,000 (MS) and 35,000 (dd-MS²). The scan range was 200–2000 m/z, with an AGC target of 1e6 (200 ms, MS; 1e6 (300 ms), dd-MS²), TopN set to 8, and an isolation window of 1.0 m/z.

Data processing was performed by Cayman Chemical Company using Lipostar software (v. 1.3.1b18; Molecular Discovery) for feature detection, noise and artifact reduction, alignment, normalization and lipid identification. Heatmaps were generated using MetaboAnalyst 6.0 [72].

Raw data are provided as Supplementary Files 2, 3.

### Western blotting

Cell lysates were collected using RIPA buffer (Sigma-Aldrich, R0278) with protease and phosphatase inhibitors on ice. Protein lysates were quantified using the Pierce^TM^ BCA Protein Assay Kit (Thermo Scientific, 23227). Lysates were diluted in loading dye (Invitrogen, NP0007), reducing agent (Invitrogen, NP0009), and RIPA buffer to a protein concentration of 20 ug per sample prior to loading onto a NuPAGE 4–12% Bis-Tris gel (Invitrogen, NP0336BOX) for electrophoresis at 150 V for 45 min in NuPAGE MOPS SDS Running Buffer (Invitrogen, NP0001). SeeBlue Plus2 (Invitrogen, LC5925) was used as a protein ladder. Protein was then blotted to a methanol-activated PVDF membrane (Millipore, IPFL00010) in NuPAGE transfer buffer (Invitrogen, NP00061) at 25 V for one hour. Membranes were incubated at room temperature in 5% nonfat milk blocking buffer (Bio-Rad, 1706404) for one hour followed by incubation in the indicated antibody overnight at 4 °C. Membranes were washed three times in tris-buffered saline (Bio-Rad, 1706435) with 0.1% Tween-20 (Sigma-Aldrich, 9005-64-5) for 5 min per wash following primary and secondary antibody incubations. Proteins were detected with Clarity Max ECL Substrate (Bio-Rad, 705062) using the Bio-Rad ChemiDoc Imaging System (Image Lab Touch Software version 2.4.0.03). Anti-GPX4 (Abcam, ab125066) and anti-Vinculin (Cell Signaling, 13901 S) primary antibodies were used at 1:1,000 dilution, and anti-rabbit-HRP (Cell Signaling, 7074 S) secondary antibody was used at 1:10,000 dilution.

Raw data are provided as Supplementary File 4.

### T cell assays

KPC7940B wild type and GPX4 knockout cells were plated at 900,000 cells per 10 cm plate in 10 mL RPMI 1640 (Gibco, 11965092) supplemented with 10% heat-inactivated FBS (Corning, 35-010-CV) for 18 h. Cells were then washed twice with 10 mL PBS and incubated in 6mL of the indicated treatment media for 4 h. Conditioned media was then collected, centrifuged, and filtered through a 0.45 μm filter. The following supplements were added to the conditioned media for culturing of CD8^+^ T cells: 1% penicillin/streptomycin (Gibco, 15140163), 50 µM 2-mercaptoethanol (Gibco, 21985023), 2 mM L-Glutamine (Gibco, 25030081), 10 mM HEPES (Gibco, 15630080), and 200 U/mL IL-2 (PeproTech, 212-12-20UG). Naïve CD8 T cells were isolated from mouse spleens and lymph nodes by magnetic bead separation (Miltenyi Biotec, 130-096-543) following the manufacturers’ protocols. CD8^+^ T cells were then seeded in a 96-well plate that was coated in a PBS solution of 1 µg/mL aCD3 (Biolegend, 100340) and 5 µg/mL aCD28 (Biolegend, 102116). T cells were then incubated for 48 h in 100 µL of their respective media, after which they were analyzed for proliferation using the CellTrace Violet Cell Proliferation Kit (Invitrogen, C34557) and activation (Millipore Sigma, MABF1556) by flow cytometry using a Bio-Rad Ze5 Cell Sorter. Data were analyzed with FlowJo v11 Software.

OT-I mice (Jackson Laboratory, strain 003831) were used in MC38-OVA T cell killing assays. The MC38 ovalbumin-expressing cells were maintained in complete RPMI 1640 medium (RPMI-1640 supplemented with 10% FBS, 1% penicillin/streptomycin, 1% L-glutamine, 10 mM HEPES, 1% nonessential amino acids, 1% sodium pyruvate, 0.1% β-mercaptoethanol and 5 ng/mL IL2). Cells were seeded in a 24-well plate at 50,000 cells per well and incubated for 24 h at 37 °C in a C02 incubator. Spleens were isolated from OT-I transgenic mice and whole splenocytes were plated in complete RPMI medium. OT-I CD8^+^ cells were activated from this culture with 5 µg/ml ovalbumin peptide (OVA257-264, InvivoGen, vac-sin) and expanded for 3 days with or without oxidized lipids (OxPL) and arachidonic acid (AA). Activated OT-I CD8^+^ cells were then added on top of the MC38-OVA cells for co-culture. Cells were stained with 7-AAD for cell death and analyzed on a flow cytometer (Cytek Aurora Spectral Analyzer). The percentage of non-viable MC38-OVA cells were calculated using cells positively stained for 7-AAD. 

### In vivo studies

#### Mice

Animal experiments were approved by the University of Michigan Institutional Animal Care and Use Committee following PRO00010606. Both C57BL/6 (strain 000664, aged 6–8 weeks) and NSG mice (strain 005557, aged 6–8 weeks) were obtained from Jackson Laboratory. Mice were housed in a pathogen-free animal facility with a 12-hour light/12-hour dark cycle, 30–70% humidity, and temperatures 20–23 °C. Mice were housed at no more than five per cage. Sample size calculations were performed in advance.

#### Prophylactic vaccination study

In each arm of this study, 10 C57BL/6J mice were used. EG7 cells were treated with either 10 µM RSL3, 10 µM Mitoxantrone, or 10 µM Oxaliplatin for 24 h, after which RSL3- and Oxaliplatin-treated cells were assessed by 7-AAD for viability and Mitoxantrone-treated cells were assessed by Trypan Blue for viability. Mice were vaccinated with a total of 1,000,000 cells per treatment condition, where RSL3-treated cells were 24% viable, Mitoxantrone-treated cells were 29% viable, and Oxaliplatin-treated cells were 28% viable. Nine days after mice received a vaccination on their left flank, mice received a subcutaneous tumor challenge of 600,000 EG7 cells on their right flank. Subcutaneous tumor injections consisted of a 100 µL cell suspension in serum-free DMEM/Matrigel (Corning, 354234) 1:1 (v/v). Tumor measurements were taken starting 10 days post-tumor challenge. For this study, all mice were taken down on day 23 post-tumor challenge, unless tumor ulcerations were observed or tumor volumes exceeded 2000 mm^3^, at which point mice were euthanized.

#### KPC7940B GPX4 knockout subcutaneous tumor study and immunohistochemistry

For both arms of this study, five C57BL/6J mice were used. Four hours prior to their preparation for subcutaneous injection, KPC7940B GPX4 KO cells were withdrawn from their standard culture conditions of DMEM supplemented with 2 µM Fer-1 and 10% FBS and grown in standard DMEM with 10% FBS for 4 h. The purpose of this step was to induce ferroptotic cell death as performed in other experiments herein. Subcutaneous tumor injections contained a total of 500,000 KPC7940B GPX4 KO cells along with an equivalent number of KPC7940B wild type cells as a control. Subcutaneous tumor injections consisted of a 100 µL cell suspension in serum-free DMEM/Matrigel (Corning, 354234) 1:1 (v/v). Tumor measurements were taken starting three days after setting subcutaneous tumors. At endpoint, tumors were fixed in 10% neutral buffered formalin for 48 h, then embedded in paraffin to create formalin-fixed paraffin-embedded FFPE blocks. Serial sections, each 4 μm thick, were cut from the FFPE blocks. Sections were deparaffinized in Histoclear and rehydrated through a graded ethanol series to distilled water. Slides were stained in hematoxylin, rinsed in tap water, and differentiated in acid alcohol if necessary. Following bluing in tap water, sections were counterstained in eosin, dehydrated through graded alcohols, cleared in Histoclear, and mounted with coverslips using Toluene. For immunostaining, sections were subjected to antigen retrieval using citrate buffer (pH 6.0) or Tris-EDTA buffer (pH 9.0) at 95–100 °C, followed by cooling to room temperature. Endogenous peroxidase activity was quenched with 3% hydrogen peroxide. After blocking with 5% normal serum, sections were incubated overnight at 4 °C with primary antibodies against GPX4 (Abcam, ab125066), CK19 (Developmental Studies Hybridoma Bank, TROMA-III), CD3 (eBioscience, 100340), F4/80 (eBioscience, 15480182), or αSMA (Millipore-Sigma, A5228) at manufacturer-recommended dilutions. The following day, slides were washed and incubated with appropriate biotinylated secondary antibodies for 30 min at room temperature. Signal was visualized using a DAB substrate kit, and sections were counterstained with hematoxylin, dehydrated, cleared, and mounted.

#### Ferroptosis-killed tumor inoculation and CyTOF analysis

In this study, 10 C57BL/6 mice were used in the control and RSL3 arms, and seven mice were used in the Mitoxantrone arm. KPC7940B cells were treated with either vehicle (control), 10 µM RSL3, or 10 µM Mitoxantrone for 24 h, after which cells were assessed by Trypan Blue for viability. Subcutaneous injections were prepared such that RSL3- and Mitoxantrone-treated arms received a total of 1,000,000 cells, where RSL3-treated cells were 27% viable and Mitoxantrone-treated cells were 29% viable. Injections for the control arm were prepared to have 250,000 viable cells. Subcutaneous tumor injections consisted of a 100 µL cell suspension in serum-free DMEM/Matrigel (Corning, 354234) 1:1 (v/v). Tumor measurements were taken starting 9 days after setting subcutaneous tumors. All mice were taken down 24 days post-tumor challenge. At endpoint, three tumors from each condition were analyzed by Cytometry by Time-of-Flight analysis. The processing of tissues and data analysis closely followed protocols that were previously described in detail [73]. The following antibodies from Standard BioTools were used at listed dilutions: CD45, 1:200 (3089005C); CD11b, 1:300 (3148003B); CD206, 1:200 (3169021B); CD3, 1:100 (3152004B); CD8, 1:200 (3168003B); CD11c, 1:100 (3209005B); Ly6c, 1:400 (3150010B); Ly-6G, 1:400 (3141008B); iNOS, 1:100 (3161011B); Arg1, 1:100 (3164027D); F4/80, 1:100 (3146008B); CD4, 1:200 (3145002B).

MC38 cells were grown in RPMI-1640 medium supplemented with 10% FBS and 1% penicillin/streptomycin. MC38 cells (500,000 or 1,000,000 cells) were treated with or without RSL3 for 24 h and then resuspended in PBS and subcutaneously injected into both flanks of C57BL/6 mice. At 20–25 days, when tumors reached an advanced stage, the mice were euthanized, and the tumors were isolated. The tumor weight/mass for each condition was measured and recorded using a Compact Scale.

#### Ferroptosis-killed tumor inoculation in BL/6 vs. NSG mice with flow cytometry analysis

For this study, three conditions—Vehicle (control), RSL3, or freeze/thaw—were tested in both immunocompetent C57BL/6 and immune incompetent NOD *scid* gamma (NSG) mice. For the C57BL/6 arm, there were 10 mice per condition, and in the NSG arm, there were five mice per condition. Subcutaneous tumor injections in the freeze/thaw condition consisted of KPC7940B cells subjected to two freeze/thaw cycles. For the other two conditions, KPC7940B cells were treated with either vehicle (control) or 10 µM RSL3 for 24 h. Subcutaneous tumor injections were prepared such that RSL3-treated, and freeze/thaw arms received a total of 1,000,000 cells, where RSL3-treated cells were approximately 30% viable and freeze/thaw-treated cells were approximately 30% viable as determined by Trypan Blue. Tumor injections for the control arm were prepared to have 300,000 viable cells. Subcutaneous tumor injections consisted of a 100 µL cell suspension in serum-free DMEM/Matrigel (Corning, 354234) 1:1 (v/v). Tumor measurements were taken starting eight days after setting subcutaneous tumors. All mice were taken down 24 days post-tumor challenge. At end point, tumors from each condition were analyzed by flow cytometry. Tumors were mechanically dissociated using scissors in sterile PBS, followed by centrifugation and resuspension in 1 mg/mL Collagenase V (Sigma) for enzymatic digestion at 37 °C for 30 min. Digestion was quenched by adding DMEM supplemented with 10% FBS. After sequentially filtering the cell suspension through 500 μm, 100 μm, and 40 μm strainers to obtain a single-cell suspension, thered blood cells were lysed, and the remaining cells were washed with PBS. Cells were then blocked and stained with antibodies in FBS. After staining, cells were washed in FACS buffer (1% Bovine Serum Albumin and 1 mM ethylenediaminetetraacetic acid in PBS) and analyzed on a MoFlo Astrios flow cytometer (Beckman Coulter). The following antibodies were used: MHCII (eBioscience,11532181), CD45.1 (eBioscience, 11045381), CD8a (BD Biosciences, 557654), CD11b (BD Biosciences, 557657), F4/80 (eBioscience, 15480182), CD11c (eBioscience, 562782), TCRb (eBioscience, 12596182).

### Statistical analysis

Data are presented as the mean ± SD from technical or biological replicates as indicated in legends. Statistical analyses were performed using GraphPad Prism 10.5.0 (GraphPad Software Inc.). Differences between experimental and control groups were assessed for significance using unpaired t-tests with Welch’s correction. Where comparisons are made among multiple experimental groups and controls, one-way analysis of variance (ANOVA) was used. A p-value below 0.05 was considered statistically significant.

## Results

### Metabolic screen identified that cysteine dropout induces features of immunogenic cell death

The objective of this study was to identify metabolic mechanisms that regulate ICD in cancer. We selected a panel of syngeneic murine tumor cell lines representing diverse cancer types. Syngeneic cell lines are genetically identical to inbred mouse strains and can be transplanted into immune competent mice without rejection. This enables the study of immune-tumor interactions in vivo. With this panel, we evaluated the role of perturbed amino acid metabolism in the induction of ICD.

**Fig. 1:**
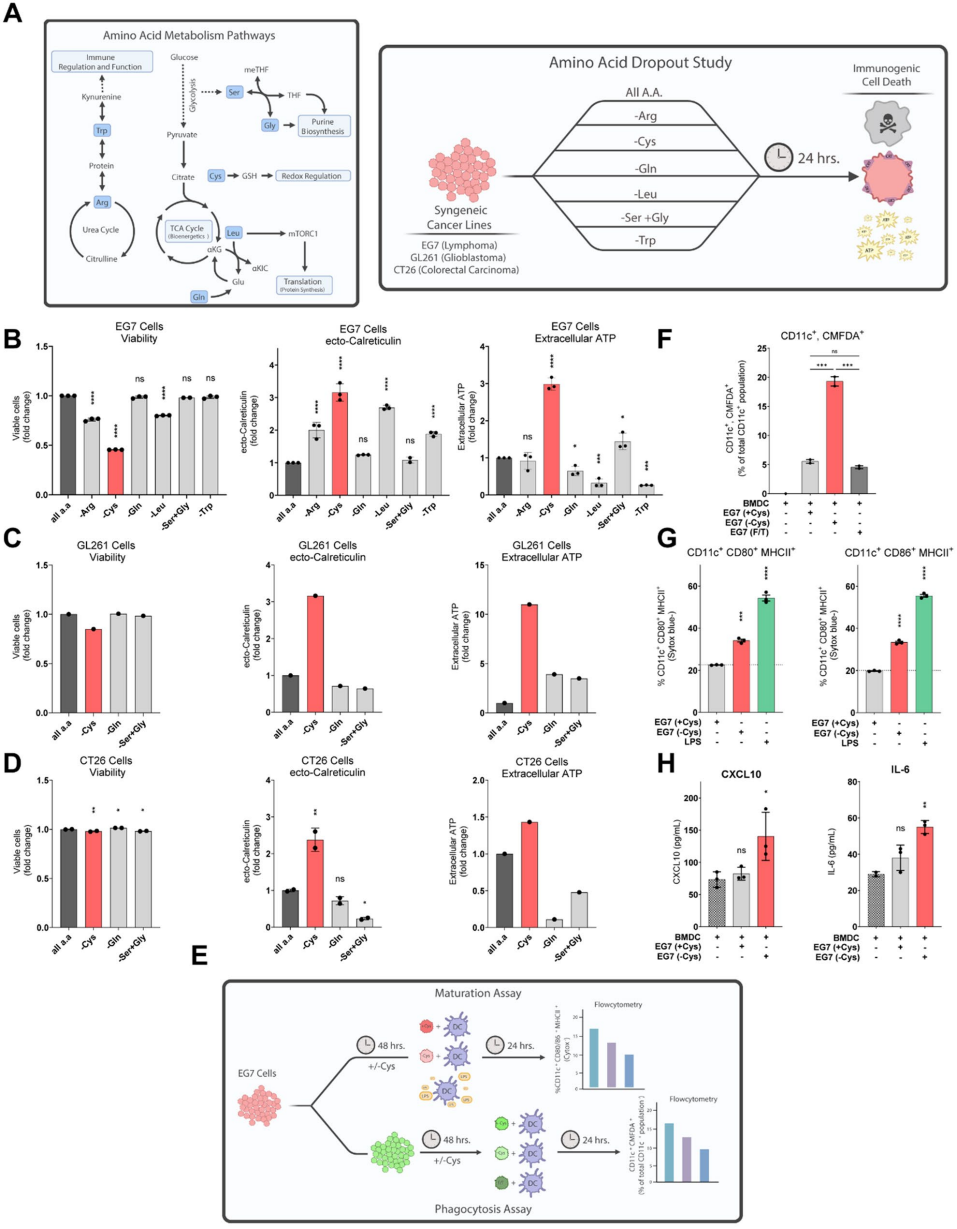
Cysteine depletion results in features of immunogenic cell death. **A**, Scheme of the amino acid metabolism pathways targeted in single amino acid dropout studies and the experimental setup of those studies. **B-D**, Viability, ecto-calreticulin, and extracellular ATP measured in EG7 (**B**), GL261 (**C**), and CT26 (**D**) cell lines at 24 h under single amino acid dropout conditions. **E**, Experimental scheme depicting maturation and phagocytosis assays with murine BMDMs. **F**,** G**, Flow cytometry analysis of murine BMDCs assessing (**F**) phagocytosis of co-incubated EG7 cells, as well as (**G**) maturation markers CD80, CD86, and MHCII. EG7 pretreatment conditions: Full RPMI (+ Cys), cystine-free RMPI (-Cys), and freeze/thaw (F/T). **H**, ELISA of supernatant from phagocytosis assay measuring CXCL10 and IL-6. Individual data points are presented over bar graphs with error bars, which represent the mean ± SD of technical replicates, where ns is not significant, *P* ≥ 0.05; *P < 0.05; **P < 0.01; ***P < 0.001; ****P < 0.0001

To select feasible amino acid metabolism pathways to target, we identified several promising amino acids that are druggable, diet-amendable, experimentally tractable, and have known roles in cell death or immunity. Our target list included: tryptophan (Trp) and arginine (Arg), due to their regulation of immune function; glutamine (Gln) and Leucine (Leu) for their proximity to mTORC1 signaling and subsequent roles in selective protein synthesis; serine (Ser) and glycine (Gly), due to their essentiality for purine biosynthesis; and cysteine (Cys) for its role in redox regulation (Fig. 1A). EG7, CT26, and GL261 cells were cultured for 24 h in media with dialyzed FBS individually depleted of each of these amino acids, after which cell viability and markers of ICD were quantified. Dropout of cysteine elicited the highest levels of DAMPs, measured by ATP release into the culture media and calreticulin exposure on the cell surface (ecto-calreticulin), among individual amino acid dropouts, prompting us to further explore cysteine as a regulator of ICD (Fig. 1B-D).

After measuring cell-autonomous ICD features, we next sought to assess whether cysteine restriction in EG7 cells is sufficient to initiate an immune response. Dendritic cells sit at the crossroads of adaptive and innate immunity and are required for anti-tumor immune activity [74, 75]. To effectively prime cytotoxic T cells via antigen presentation, both dendritic cell phagocytosis and maturation are essential [76–79]. To assess these processes, we established co-cultures of cysteine-deprived EG7 cells and bone marrow derived dendritic cells (BMDC). In these studies, EG7 cells were grown in cysteine-deprived or replete conditions for 48 h prior to coculture with BMDCs (Fig. 1E). BMDC phagocytosis of EG7 cells was greater in co-cultures with cysteine-deprived EG7 cells than in co-cultures with cysteine replete cells or cells undergoing non-immunogenic death (freeze-thaw) (Fig. 1F, Supp Fig. 1A). Additionally, markers of BMDC maturation (CD80, CD86, and MHCII [80]) and function (proinflammatory cytokines IL-6 and CXCL10) were enhanced in co-cultures with cysteine-deprived EG7 cells (Fig. 1G, H). Altogether, these data demonstrate that cysteine restriction induces DAMP signaling by EG7 cells, which subsequently promotes dendritic cell phagocytosis, maturation, and cytokine production.

### GPX4 inhibition-induced ferroptosis promotes features of immunogenic cell death in PDAC

**Fig. 2:**
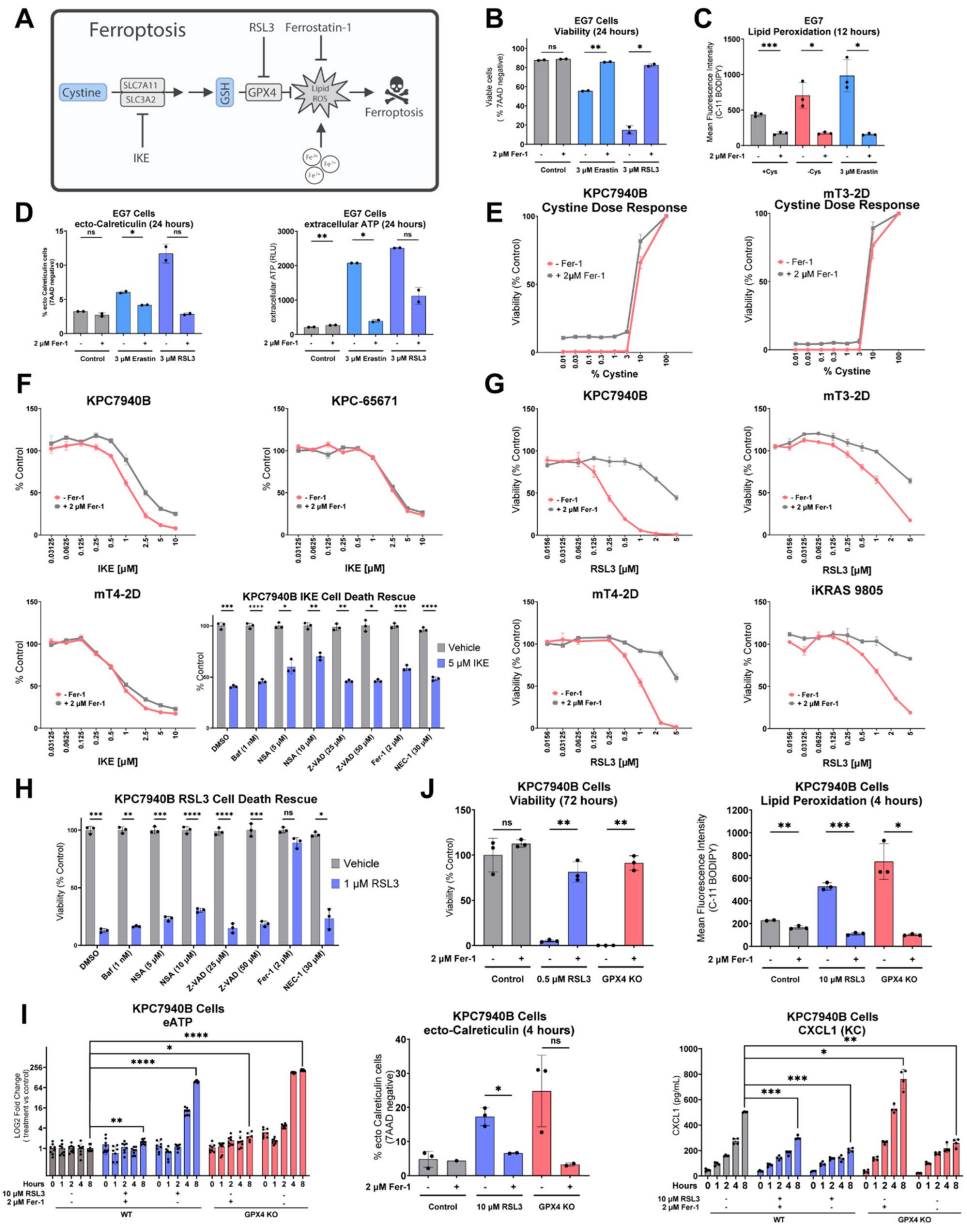
GPX inhibition-induced ferroptosis in PDAC cells results in features of immunogenic cell death. **A**, Scheme depicting canonical ferroptosis pathway. **B**, Viability of EG7 cells after 24-hour treatment with 3 µM RSL3 or 3 µM erastin, +/- 2 µM Fer-1. **C**, Lipid peroxidation of EG7 cells after 12-hour incubation with complete RPMI (+ Cys), RPMI without Cystine (-Cys), or 3 µM erastin in complete RMPI, each condition +/- 2 µM Fer-1. **D**, ecto-Calreticulin positive EG7 cells and the ATP measured in their media after 24-hour treatment with 3 µM RSL3 or 3 µM erastin, each condition +/- 2 µM Fer-1. **E**, Viability of KPC7940B and mT3-2D cells grown under varied concentrations of cystine for 96 h +/- 2 µM Fer-1, where [cystine] at 100% = 0.0652 g/L (comparable to complete RPMI). **F**, 48-hour dose response curves of imidazole ketone erastin (IKE)-treated KPC7940B, KPC65671, mT4-2D cells +/- 2 µM Fer-1. (below) Viability of KPC7940B cells after 48-hour co-treatment with IKE and various cell death inhibitors. **G**, 48-hour dose response curves of RSL3-treated KPC7940B, mT3-2D, mT4-2D, and iKRAS 9805 cells +/- 2 µM Fer-1. **H**, Viability of KPC7940B cells after 48 h of co-treatment with 1 µM RSL3 and various cell death inhibitors. **I**, (left) ATP and (right) CXCL1 measured in the media of 10 µM RSL3-treated or GPX4-deleted KPC7940B cells +/- 2 µM Fer-1 at time points from 0–8 h. (middle) ecto-Calreticulin positive KPC7940B cells after 4-hour treatment with 10 µM RSL3 or withdrawal of 2 µM Fer-1 from GPX4-deleted KPC7940B cells. **J**, Viability of KPC7940B cells after 72-hour treatment with 0.5 µM RSL3 or after 72-hour withdrawal of 2 µM Fer-1 from GPX4-deleted KPC7940B cells. Lipid peroxidation measured in KPC7940B cells after 12-hour treatment with 10 µM RSL3 or 12-hour withdrawal of 2 µM Fer-1 from GPX4-deleted KPC7940B cells. Individual data points are presented over bar graphs with error bars, which represent the mean ± SD of technical replicates, where ns is not significant, *P* ≥ 0.05; *P < 0.05; **P < 0.01; ***P < 0.001; ****P < 0.0001

Ferroptosis is a metabolic form of regulated cell death involving amino acid, iron, and lipid metabolism that results from uncontrollable lipid oxidation and cell rupture [81–89]. Prompted by our findings that EG7 cells respond to cysteine deprivation by initiating ICD, and given the well-appreciated role of cysteine in ferroptosis (Fig. 2A), we next investigated whether the same induction of ICD features is observed during ferroptotic cell death.

Cystine restriction and inhibition of cysteine import are well established inducers of ferroptosis [90]. Indeed, EG7 cell death by cysteine depletion was rescued by the lipid peroxyradical trapping drug Ferrostatin-1 (Fer-1) [91], a phenomenon similarly observed in cells treated with erastin, an inhibitor of cysteine import through system x_c_^−^ [92] (Fig. 2B, Supp Fig. 1B-C). To further interrogate ferroptosis, we measured lipid reactive oxygen species (ROS), the ultimate mediator of ferroptosis. Comparable to erastin treated cells, cysteine-deprived EG7 cells demonstrated lipid peroxidation that was rescued by Fer-1 (Fig. 2C). Consistent with our initial cysteine deprivation assays, 24-hour treatment with erastin induced DAMPs, extracellular ATP and ecto-calreticulin, in EG7 cells, and this was rescued by Fer-1 (Fig. 2D, Supp Fig. 1D). These results were also recreated by treatment with RSL3, an inhibitor of the antioxidant protein glutathione peroxidase 4 (GPX4) that prevents ferroptotic cell death by detoxifying oxidized phospholipids [83, 89, 93, 94] (Fig. 2A-B and D). Together, these results suggest that the immunogenic properties of cysteine deprivation result from ferroptotic cell death.

In syngeneic PDAC cells (KPC7940B, mT3-2D, KPC-65671), cystine deprivation through media dropout or system x_C_^−^ inhibition with imidazole ketone erastin (IKE) leads to cell death; however, this was not meaningfully rescued by Fer-1 in vitro, suggesting that cysteine restriction causes cell death through a mechanism independent of ferroptosis (Fig. 2E, F). In contrast, RSL3-induced cell death was rescued by Fer-1 in four syngeneic murine PDAC cell lines (Fig. 2G). Furthermore, using a panel of autophagy, necrosis, apoptosis, and necroptosis inhibitors, only Fer-1 conferred a rescue of RSL3-induced cell death, reflecting ferroptosis as the mediator of RSL3-induced cell death in our PDAC model (Fig. 2H).

Thus, for subsequent PDAC studies, we investigated the immunogenicity of GPX4-inhibition-induced ferroptosis. To this end, we used RSL3 alongside a GPX4 knockout cell line propagated in Fer-1 supplemented media (Supp Fig. 1E). We chose the KPC7940B cell model due to its sensitivity to RSL3-mediated GPX4 inhibition and robust rescue by Fer-1 (Fig. 2G, Supp Fig. 1F, G). Induction of ferroptosis by RSL3 treatment or restriction of Fer-1 from GPX4 knockout cells led to cell death and lipid peroxidation that could be rescued by Fer-1 (Fig. 2J). Importantly, induction of ferroptosis by RSL3 treatment or restriction of Fer-1 from GPX4 knockout cells also lead to the rapid release of ATP, exposure of ecto-calreticulin, and the suppression of CXCL1 secretion [61] (Fig. 2I). The induction of these DAMPs was blocked when cells were co-treated with Fer-1.

Characteristics of ferroptotic cell death have been previously divided into three time-dependent stages [93–98]. During initial ferroptosis (0–2 h after the induction of ferroptosis), cells begin to accumulate lipid ROS. This is followed by an intermediate stage (3–4 h), characterized by partial permeabilization of the cell membrane, leading to the release of ATP and the exposure of calreticulin. Finally, in terminal ferroptosis (6–8 h), complete membrane permeabilization occurs, resulting in the release of LDH, HMGB1, and various cytokines. Our results demonstrating a temporal release of ATP, ecto-calreticulin exposure, and dampened CXCL1 secretion by ferroptotic cells coincided closely to these stages (Fig. 2I). Taken together, our data illustrate that ferroptosis initiated by GPX4 inhibition or knockout leads to features consistent with an immunogenic form of cell death.

### GPX4 inhibition-induced ferroptosis in PDAC cells leads to the selective release of immunosuppressive metabolites and lipids

Next, to identify metabolite-derived immunomodulatory signals, we set out to profile intra- and extracellular metabolomes and lipidomes by mass spectrometry. Informed by the time course described above, we selected a four-hour time point for the metabolomic and lipidomic analyses. Here, we employed two orthogonal models of ferroptosis: RSL3-treated wild type KPC7940B cells and Fer-1-withdrawn GPX4 knockout KPC7940B cells. We compared the relative abundance of individual metabolites in cells and media supernatants under basal and ferroptotic conditions. To screen for specific metabolites released by cells in early or intermediate ferroptosis, rather than bulk content from cells in terminal ferroptosis with ruptured plasma membranes, we used extracellular levels of glycolytic intermediates across conditions as a surrogate for membrane rupture. Phosphorylated compounds are generally not secreted from cells, with exceptions for some transported metabolites (Supp Fig. 1H-I).

**Fig. 3:**
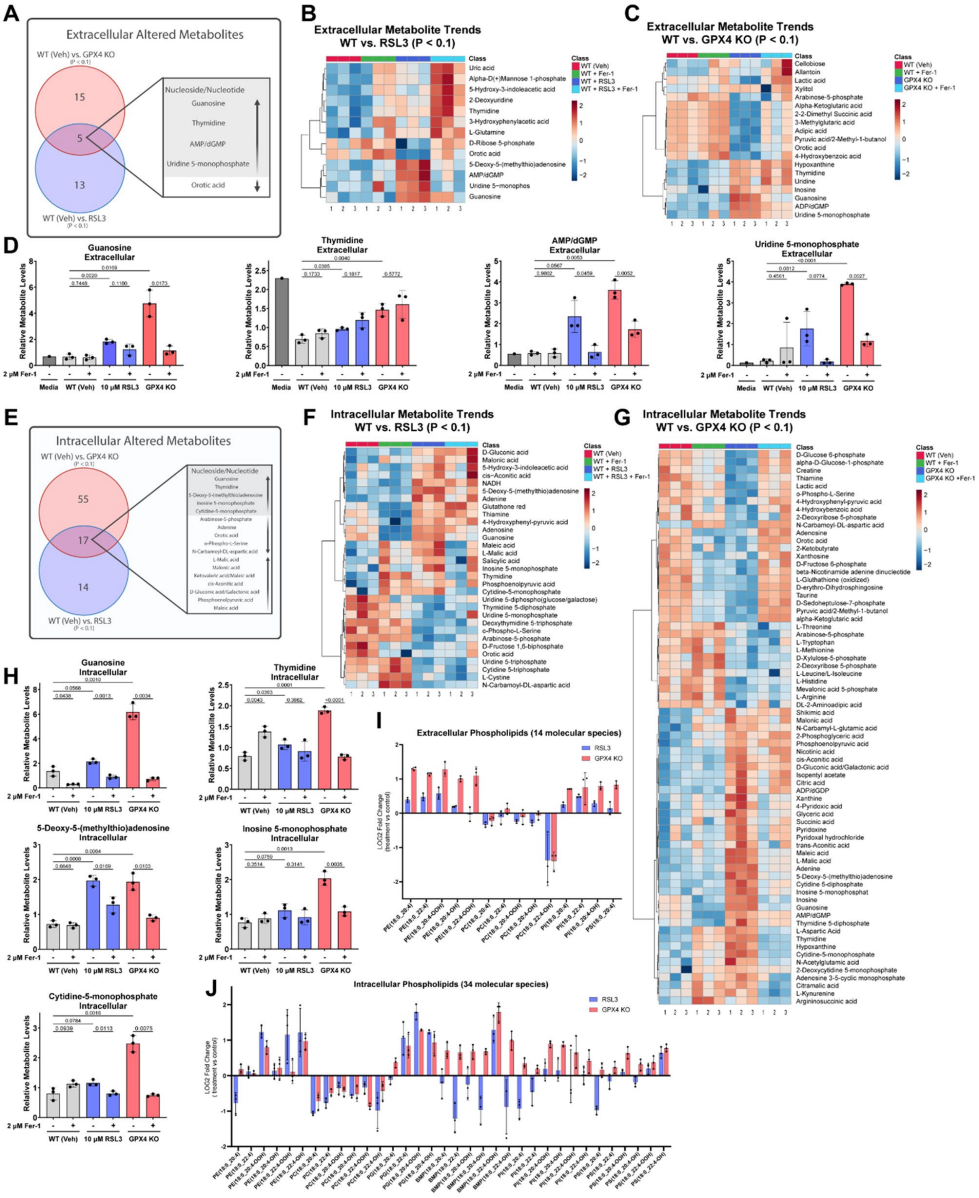
GPX4 inhibition-induced ferroptosis in PDAC cells results in the selective secretion of immunosuppressive metabolites. **A**, Scheme of metabolomics study showing consistently changing metabolites in the culture media of RSL3-treated and GPX4 KO KPC7940B cells, relative to vehicle. **B**, Heatmap showing differentially changed (*P* < 0.1) metabolites in the media supernatants of RSL3-treated and WT (Veh) KPC7940B cells **C**, or differentially changed (*P* < 0.1) metabolites in media supernatants of GPX4 KO and WT (Veh) KPC7940B cells. Log10 fold change is shown. **D**, Relative abundance of Guanosine, Thymine, Adenosine 5-monophosphate/2-Deoxyguanosine 5-monophosphate (AMP/dGMP), and Uridine 5-monophosphate in media supernatants of WT (Veh), RSL3-treated, and GPX4 KO KPC7940B cells, each with and without Fer-1 treatment. **E**, Scheme of metabolomics study showing consistently changing metabolites in the cell pellets of RSL3-treated or GPX4 KO KPC7940B cells. **F**,** G**, Heatmap showing differential (*P* < 0.1) metabolites in the cell pellets of RSL3-treated and WT (Veh) KPC7940B cells (**F**) or differential (*P* < 0.1) metabolites in cell pellets of GPX4 KO and WT (Veh) KPC7940B cells (**G**). Log10 fold change is shown. **H**, Relative abundance of Guanosine, Thymidine, 5-Deoxy-5-(methylthio)adenosine, Inosine 5-monophosphate, and Cytidine-5-monophosphate in cell pellets of WT (Veh), RSL3-treated, and GPX4 KO KPC7940B cells, each with and without Fer-1 treatment. **I**, Log2 fold change of oxidized phospholipids detected by targeted mass spectrometry in supernatants of RSL3-treated and GPX4 KO KPC7940B cells as compared to WT (Veh) control. **J**, Oxidized phospholipids detected by targeted mass spectrometry in cell pellets of RSL3-treated and GPX4 KO KPC7940B cells as compared to WT (Veh) control. Individual data points are presented over bar graphs with error bars, which represent the mean ± SD of technical replicates, where ns is not significant, *P* ≥ 0.05; *P < 0.05; **P < 0.01; ***P < 0.001; ****P < 0.0001

Media supernatants from RSL3-treated and GPX4 knockout cells revealed a consistent increase in the secretion of select nucleosides and nucleotides, whose abundance could be reversed when ferroptosis was inhibited with Fer-1 (Fig. 3A-D). A broader range of similarly regulated metabolites was observed in cell pellets of RSL3-treated and GPX4 knockout cells, with a distinct signature suggesting nucleoside and nucleotide metabolism in ferroptotic cells (Fig. 3E-H). Of the metabolites that we identified in extracellular fractions of ferroptotic cells, adenylates (e.g. ATP and AMP) are most notably reported for their ability to modulate immune activity [95–100]. Adenosine, derived from ATP and AMP in the TME through a series of conversions mediated by CD39 and CD73 [101–107], has been well-characterized as a general immunosuppressive metabolite [108–122]. In addition, 2-Deoxyguanosine 5-monophosphate (dGMP) has been reported to suppress the secretion of IFN-γ from peripheral blood mononuclear cells when co-cultured with viral antigen [123].

Next, we analyzed the intra- and extracellular lipidomes of ferroptotic cells. Lipid peroxidation acts as a central driver of ferroptotic cell death, with the accumulation of oxidized polyunsaturated phospholipids leading to membrane damage, cell rupture, and ultimately cell death [124–126]. During ferroptosis, the most well-reported oxidized phospholipids are polyunsaturated phospholipids containing phosphatidylethanolamines (PEs), particularly those with arachidonic acid (AA, C20:4) and adrenic acid (AdA, C22:4) side chains [127–130]. In addition to oxidized PEs, certain oxidized phosphatidylcholines (PCs), such as 1-palmitoyl-2-(5-oxovaleroyl)-sn-glycero-3-phosphocholine (POVPC) and 1-palmitoyl-2-glutaryl-sn-glycero-3-phosphocholine (PGPC) [131], have also been detected during ferroptosis and are known to contribute to membrane damage and cell death [132]. While oxidized forms of other phospholipids like phosphatidylserine (PS) and phosphatidylinositol (PI) have been observed, their roles are less well characterized compared to PE and PC species. Using a parallel scheme as to that of our metabolomic analysis, LC-MS lipidomic analysis focused on oxidized phospholipids revealed elevated levels of phospholipid hydroperoxide in both intra- and extracellular fractions of ferroptotic cells (Fig. 3I, J, Supp Fig. 2A-B). Untargeted LC-MS showed similar trends of oxidized phospholipids between RSL3-treated and GPX4 knockout cells in media supernatants (Supp Fig. 2C-D, Supp Fig. 3A-D). Taken in whole, these lipidomic data are consistent with previous reports and suggest that lipids are a potential source of immunomodulatory signals emitted by ferroptotic PDAC cells.

### Oxidized lipids inhibit CD4^+^ and CD8^+^ proliferation and effector function

**Fig. 4:**
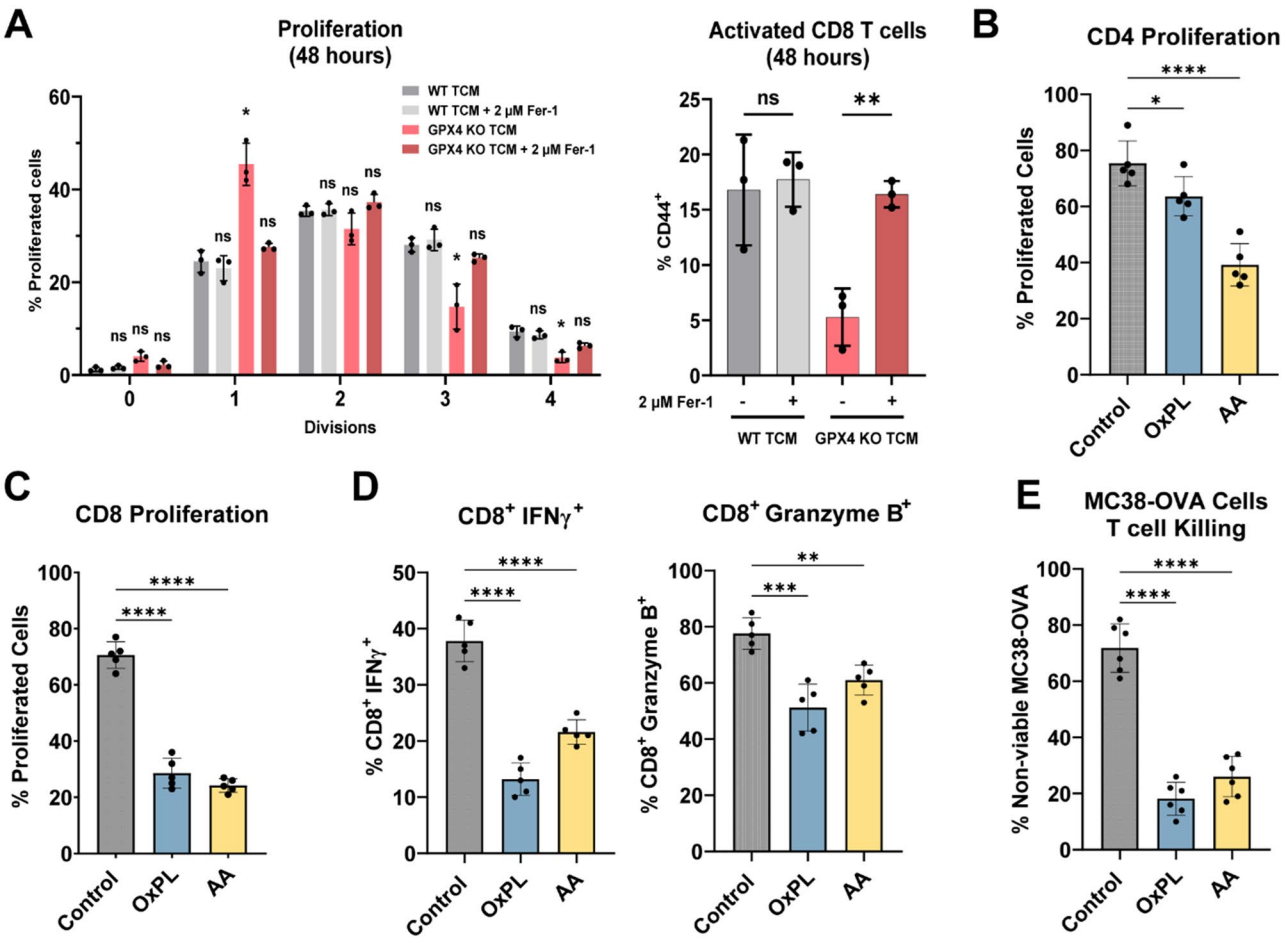
Oxidized lipids inhibit CD4^+^ and CD8^+^ proliferation and effector function. **A**, (left) Proliferation of CD8^+^ T cells cultured in conditioned media from WT KPC7940B cells grown in +/- 2 µM Fer-1 media or conditioned media from GPX4-deleted KPC7940B cells grown in +/- 2 µM Fer-1 media. (right) In parallel, the percentage of CD44^+^ CD8^+^ T cells was measured for these conditions. **B**, Proliferation of CD4^+^ and **C**, CD8^+^ T cells cultured in oxidized lipids (OxPL) or arachidonic acid (AA). **D**, Measurement of CD8^+^ IFNγ^+^ and CD8^+^ Granzyme B^+^ T cells cultured in OxPL or AA. **E**, T cell killing assay measuring the viability of MC38-OVA cells cocultured with OT-1 CD8^+^ T cells. OT-1 CD8^+^ T cells were preincubated in either OxPL or AA before coculture with MC38-OVA cells. Individual data points are presented over bar graphs with error bars, which represent the mean ± SD of technical replicates. Data in **D-E** represent technical replicates from two biologically independent samples. ns is not significant, *P* ≥ 0.05; *P < 0.05; **P < 0.01; ***P < 0.001; ****P < 0.0001

We next assessed the effect of PDAC ferroptosis conditioned media on CD8^+^ T cell activation. Using conditioned media from KPC7940B GPX4 knockout cells undergoing intermediate ferroptosis, we observed that CD8^+^ T cell proliferation and activation (as indicated by CD44 expression) were suppressed, phenotypes not observed in either wild type cells or GPX4 knockout cells cultured in Fer-1 (Fig. 4A, Supp Fig. 4A). Guided by our extracellular lipidomics data, we hypothesized that the inhibitory property of ferroptotic cell condition media was a consequence of oxidized lipids. To test this hypothesis, we assessed the impact of an oxidized phospholipids (OxPL) pool or arachidonic acid (AA) directly on CD4^+^ and CD8^+^ T cells. AA spontaneously oxidizes in culture, serving as a defined alternative to OxPL. Indeed, OxPL and AA both inhibited CD4^+^ and CD8^+^T cell proliferation and CD8^+^ T cell effector molecule production (i.e. IFNγ and Granzyme B) (Fig. 4B-D). To directly test T cell functionality, we generated CD8^+^ T cells from OT-I mice, which recognize the ovalbumin antigen (OVA). By engineering MC38 syngeneic murine colon cancer cells to express OVA, we tested the ability of CD8^+^ T cells to kill cancer cells. Indeed, pretreatment of activated OT-I T cells with OxPL or AA significantly diminished their ability to kill cancer cells (Fig. 4E). These data illustrate that oxidized lipids impair CD8^+^ T cell proliferation, activation, and cytotoxic. Our evidence has thus far revealed both immunogenic (i.e. DAMP release, dendritic cell activation) and immunosuppressive (i.e. oxidized metabolite and lipid release, suppression of T cells) features of ferroptotic cell death in PDAC cells. To understand the balance of these opposing activities on immune function in the tumor, we next turned to in vivo models.

### GPX4-inhibition promotes subcutaneous tumor outgrowth in tumor challenge experiments by altering immune populations

**Fig. 5:**
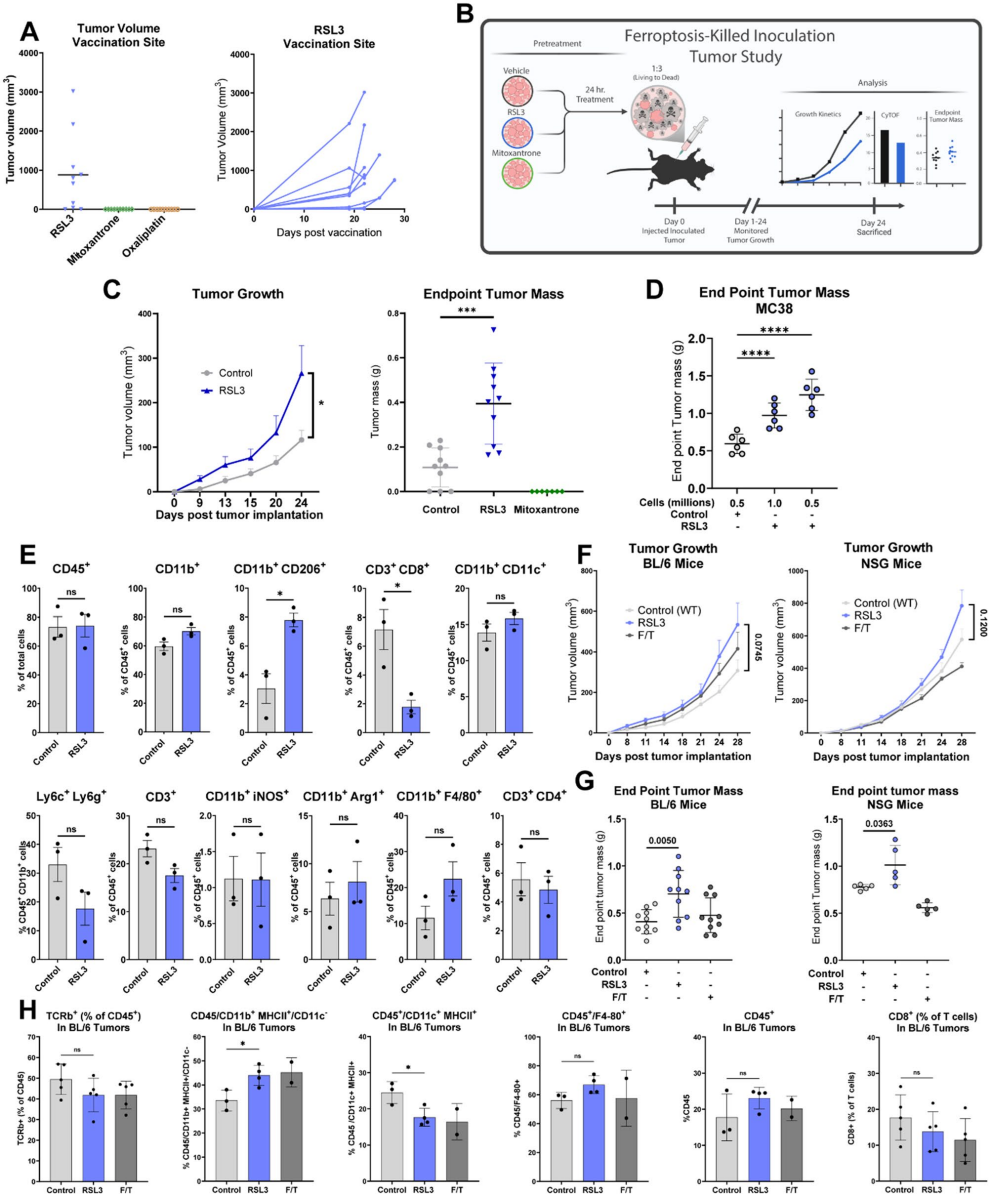
Inhibiting GPX4 in PDAC cells alters immune populations and enhances subcutaneous tumor outgrowth. **A**, (left) Endpoint subcutaneous tumor volume at vaccination site 22 days post prophylactic vaccination with 76% RSL3-killed, 71% mitoxantrone-killed, or 72% oxaliplatin-killed EG7 cells. Pre-injection cell viability was determined by flow cytometry. Mice vaccinated with mitoxantrone- and oxaliplatin-killed cells grew no tumors at their vaccination sites. All mice vaccinated with RSL3-killed cells were tumor-bearing at endpoint. (right) Subcutaneous tumor volume measured at the vaccination site in mice given RSL3-killed tumor vaccinations. **B**, Scheme of ferroptosis-killed tumor inoculation study with PDAC cells. **C**, (left) Subcutaneous tumor volume and (right) end point tumor mass of mice given a subcutaneous injection of living and RSL3-killed KPC7940B cells or a subcutaneous injection of an equivalent number of living cells. Tumors did not grow in mice given subcutaneous injections containing mitoxantrone-killed KPC7940B cells. **D**, End point tumor mass in BL/6 mice given subcutaneous injections of viable and RSL3-killed MC38 cells (RSL3) or viable MC38 cells alone (control). **E**, CyTOF analysis of subcutaneous tumors from three separate mice in RSL3 and Control arms from (**C**). **F**, Tumor volume measured across time and **G**, end point tumor mass in BL/6 and NSD mice given a subcutaneous injection of living and RSL3-killed KPC7940B cells (1:3) or living (RSL3) and freeze/thaw-killed KPC7940B cells (F/T) (1:3). Mice in the control arm (Control (WT)) were given a subcutaneous injection with an equivalent number of living KPC7940B cells. **H**, Flow cytometry analysis of subcutaneous tumors from separate mice in control, RSL3, and freeze/thaw tumor injection conditions from BL/6 mice in (**F**,** G**). Individual data points are presented over bar graphs with error bars, which represent the mean ± SD of technical replicates, where ns is not significant, *P* ≥ 0.05; *P < 0.05; **P < 0.01; ***P < 0.001; ****P < 0.0001

The gold standard to evaluate the immunogenicity of cell death in vivo is the prophylactic vaccination of immunocompetent mice with pre-killed cancer cells, followed by a challenge with healthy malignant cells [125]. To attempt this using ferroptotic cells, we vaccinated mice with EG7 cells pre-treated with an IC90 dose of RSL3 or positive control chemotherapies (mitoxanthrone or oxaliplatin). Unexpectedly, upon initial vaccination, cells pre-treated with RSL3, but not with either chemotherapy, grew to establish tumors at the vaccination site (Fig. 5A). This suggested that ferroptotic signaling from RSL3-treated cells coordinates a protective environment that facilitates tumor growth at the vaccination site. To address this and evaluate an alternative means of vaccination, we tested the ability of GPX4 knockout cells lacking Fer-1 to grow in vivo. Surprisingly, these cells were able to form small tumors (Supp Fig. 4B-C), a reversal of our in vitro results highlighting the need for Fer-1 to maintain the viability of GPX4 knockout cells (Supp Fig. 1E). Given that both of these prophylactic vaccination strategies resulted in tumor outgrowth at the vaccination site, potentially interfering with the interpretation of results from a subsequent challenge with healthy cells, we instead turned to a live/dead mixed tumor inoculation model.

To determine whether ferroptosis impacts PDAC tumor growth, we mixed wildtype KPC7940B tumor cells with RSL3-treated cells or chemotherapy-treated controls at a 1:3 living cell to dead cell ratio and implanted tumors subcutaneously (Fig. 5B). Tumors seeded with RSL3-treated cells had significantly larger mass at endpoint and increased growth kinetics compared to wildtype KPC7940B tumors (Fig. 5C). To validate these results in an additional model, we repeated this experiment using MC38 colorectal cancer cells, with tumors containing a mix of RSL3-treated cells again outperforming their untreated counterparts (Fig. 5D). Together, these data provide support for an immune-dependent role of ferroptosis in promoting tumor growth.

To interrogate the role of ferroptosis in shaping the TME, we quantified immune populations in control and RSL3 pre-treated tumors with cytometry by time-of-flight (CyTOF). This analysis revealed that RSL3-treated tumors were enriched with immunosuppressive myeloid cells (CD11b^+^/CD206^+^) and had reduced infiltration of CD8^+^ T cells (Fig. 5E, Supp Fig. 4D). To understand whether impaired CD8^+^ T cell infiltration is responsible for the enhanced growth of ferroptotic tumors, we implanted control or RSL3 pre-treated tumors into immunocompetent or immunodeficient (NSG) mice, which lack an adaptive immune compartment and natural killer cells. Growth kinetics and endpoint weights of RSL3-treated tumors were elevated relative to controls in both immunocompetent and NSG mice, suggesting that differential activity of CD8^+^ T cells alone does not account for the enhanced growth of RSL3-treated tumors (Fig. 5F-G). We observed similar effects of RSL3 treatment on the composition of the TME in immunocompetent mice as reported above, confirming that ferroptotic tumor growth was likely supported by the same mechanism (Fig. 5H). These results demonstrate that inducing ferroptosis in PDAC tumors reshapes the tumor-immune microenvironment and supports tumor growth dynamics. While we show that impairment of the anti-tumor CD8^+^ T cell response does not account for this phenomenon on its own, our data point to the importance of innate mechanisms, such as the recruitment of immunosuppressive myeloid cells.

## Discussion

In this study, we define a role for ferroptosis in promoting immunosuppressive properties of the TME. We show that murine cancer cell lines in cysteine-deprived conditions produce adjuvants indicative of ferroptotic cell death, which promote the maturation of BMDCs and phagocytosis of ferroptotic cells. We demonstrate that the induction of ferroptosis through RSL3 or genetic deletion of GPX4 results in an ICD phenotype in vitro, which is rescued by the ferroptosis-relieving drug Fer-1. Our lipid- and metabolomic analysis of RSL3-treated or GPX4 knockout PDAC cells revealed the selective release of immunosuppressive metabolites and lipids during early and intermediate ferroptosis, which could counteract the immune-stimulatory properties of DAMPs. Indeed, we found that conditioned media from ferroptotic PDAC cells impaired the proliferation, activation, and cytotoxicity of CD8^+^ T cells. To evaluate the importance of these opposing immune stimulatory and suppressive functions in tumor immunity, we turned to in vivo tumor models. Induction of ferroptosis in implanted tumor cells led to an increase of immunosuppressive myeloid cells and reduced numbers of infiltrating CD8^+^ T cells, indicating that the immunosuppressive properties of tumor cell ferroptosis outweigh immune stimulatory signaling through DAMPs in vivo. Further analysis of tumor growth in immunocompetent BL/6 and immunodeficient NSG mice ruled out the possibility that ferroptosis acts on the adaptive immune compartment alone to mediate immunosuppression. These findings suggest that on balance, the effects of ferroptotic tumor cell death help to establish a tumor-protective, immune-tolerant microenvironment in murine PDAC.

While ferroptosis has emerged as an exciting new avenue for PDAC therapy [90, 133–137], it is necessary to carefully consider its impacts on the tumor-immune microenvironment and its overall contribution to tumor growth. Previous work establishes that ferroptosis induces immune dysfunction and tumor progression. For example, phagocytosis of ferroptotic tumor cells by BMDCs has been shown to hamper their antigen-presenting capabilities and subsequently impair T cell activation [132]. Ferroptotic tumor cells have also been found to release prostaglandin E₂ [138], which polarizes macrophages toward a tumor-supportive state and activates immunosuppressive cells like myeloid-derived suppressor cells and regulatory T cells [139, 140]. In addition, lipid peroxidation products (e.g. 4-hydroxynonenal) generated during ferroptosis induce endoplasmic reticulum stress in immune cells, further exacerbating immune dysfunction [141]. Among others, these mechanisms illustrate how ferroptosis may facilitate tumor immune evasion and progression.

Conversely, T cell-driven ferroptosis has been shown to enhance the efficacy of immunotherapies like anti-PD-1 checkpoint blockade in preclinical models [142, 143]. Clinical data also correlates markers of ferroptosis with positive responses to immunotherapy, suggesting that ferroptosis may play a role in the efficacy of immune checkpoint inhibitors [142]. In murine melanoma models, SLC7A11-deficient tumors displayed increased sensitivity to combined radiotherapy and anti-PD-L1 therapy, resulting in durable immunologic memory [144]. Another study showed that tumor cells sensitized to ferroptosis by inhibition of the itaconate transporter SLC13A3 enabled the effective treatment of immune checkpoint blockade-resistant tumors [145]. These seemingly inconsistent roles of ferroptosis in immunity highlight its complex role in modulating tumor-immune interactions.

While the release of DAMPs and cytokines during ferroptotic cell death may lead to immune activation in vitro, the fates of these products are not as straightforward in vivo. For instance, ATP, an immunogenic DAMP, is readily converted to the immunosuppressive metabolite adenosine in the TME via the widely expressed ectonucleosides CD39 and CD73, leading to aggregation of adenosine in the tumor [146]. Thus, while ferroptosis may still lead to the production of adjuvants in the tumor, our observations suggest that the TME prevents these adjuvants from facilitating beneficial immune activity. Perhaps more importantly, our data supports the notion that DAMPs are not sufficient to generate lasting and productive anti-tumor immune responses. Our study instead illustrates the complexity of signals released by dying tumor cells, such as DAMPs, metabolites, and lipids, and underlines the importance of in vivo models to determine whether a mechanism of cell death is ultimately immunogenic or not.

In a recent study, Wiernicki et al. categorized ferroptotic cell death into early, intermediate, and terminal stages, showing that DAMP release was primarily a feature of intermediate and terminal stages. Coincubation of early ferroptotic fibrosarcoma cells hindered BMDC maturation and phagocytosis, while intermediate and terminal ferroptotic cells elicited robust maturation, and phagocytosis. Our study cooberates these findings and extends them to PDAC. Further work demonstrated that terminal ferroptotic fibrosarcoma cells weakened the antigen presentation capabilities of BMDCs. As is confirmed in our work and others, high levels of oxPLs are produced during ferroptotis [147] and may impair BMDC antigen presentation [148–151]. We also establish a causal link between this ferroptosis-associated lipid oxidation and impaired CD8^+^ T cell function. While we directly demonstrate that treatment with oxidized lipids suppresses T cell proliferation and cytotoxicity, future studies might gain further mechanistic insight into ferroptosis-mediated immune regulation by treating T cells with lipid-depleted conditioned media from ferroptotic cells. Due to the carryover of small molecules in conditioned media experiments (such as with RSL3 treatment), this may require the induction of ferroptosis by genetic ablation of GPX4 in tumor cells to avoid direct unintended cell death in T cells.

Our findings that ferroptotic tumor cells release nucleosides and nucleotides is in agreement with recently published studies from Yapici, et al., who demonstrate similar metabolic profiles from GPX4 knockout small cell lung carcinoma cells [138]. Their metabolomics profiling of cell pellets from inducible GPX4 knockout mouse embryonic fibroblasts revealed a strong enrichment of purine and pyrimidine derivatives in cell pellets during early-stage ferroptosis in GPX4 knockout cells. In addition, they observed the release of pyrimidine-related metabolites. Our data in syngeneic murine PDAC cells do not fully align with their reported work in *Gpx4*-deleted MEFs; however, this may be attributed to differences in metabolic regulation, tissue of origin, or other factors disprate between murine PDAC cells and MEFs during ferroptosis-induced cell death. In another related study, Li, et al. showed that prophylactic vaccination with terminal ferroptotic cells conferred protection against subsequent tumor challenge [137]. These cells were treated with the ferroptosis inducer N6F11 in vitro. In contrast, no significant protection was provided when mice were treated with a regiment of N6F11 in subcutaneous models, nor in orthotopic, syngineic allographs unless combined with anti-PD1 checkpoint therapy.

Our work complements other studies which provide evidence that ferroptotic tumor cell death within the TME results in adverse innate immune responses [152]. Li, et al., found that macrophages adopt tumor-supporting functions as a result of ferroptotic tissue damage, subsequently promoting *Kras*-driven PDAC tumorigenesis in mice [153]. Other groups have shown anti-inflammatory macrophage polarization and myeloid-derived suppressor cell recruitment due to the liberation of KRAS and HMGB1 by ferroptotic cells in murine models of PDAC [153] and mice with *Gpx4*-deficient liver tumors. Importantly, our results indicate that the impairment of CD8^+^ T cells is not the sole mechanism of ferroptosis-mediated immune suppression. CD8^+^ T cells are essential effectors of anti-tumor immunity and the targets of a variety of mechanisms that constrain productive immune responses in the tumor [154, 155]. However, our investigation revealed that ferroptotic cell death contributes to enhanced tumor growth even in mice lacking an adaptive immune compartment. Therefore, given our findings that implantation of ferroptotic tumor cells leads to an infiltration of suppressive myeloid cells in the tumor, along with previous work highlighting ferroptosis-mediated innate immune remodeling, we propose a model wherein ferroptotic signaling coordinates immune suppression independent of the adaptive immune system. To determine the role of innate immune cells, future studies should consider depleting myeloid populations, such as macrophages (via inhibition of CSF1R or clodronate liposomes) and evaluating tumor growth dynamics after the induction of ferroptosis.

## Conclusions

In total, it is important to consider the influence of the TME on both the process and effect of ICD. Our work highlights that complexity in the TME may elicit unexpected responses to ICD such as ferroptosis. Further examination into both the tumor-intrinsic and -extrinsic effects that undermine the immunogenicity of ferroptotic cell death may uncover actionable targets and unlock the therapeutic potential of ferroptosis.

## Supplementary Information

Below is the link to the electronic supplementary material.

Supplementary Material 1

Supplementary Material 2

Supplementary Material 3

Supplementary Material 4

Supplementary Material 5

## Data Availability

All data generated or analyzed during this study are included in this published article and its supplementary information files.

## Abbreviations

PDAC: Pancreatic ductal adenocarcinoma
GPX4: Glutathione peroxidase 4
Fer-1: Ferrostatin-1
TME: Tumor microenvironment
ATP: Adenosine triphosphate
DAMP: Damage associated molecular pattern
ICD: Immunogenic cell death
BMDC: Bone marrow derived dendritic cell
ROS: Reactive oxygen species
IKE: Imidazole ketone erastin
FBS: Fetal bovine serum
RPMI: Roswell Park Memorial Institute
DMEM: Dulbecco’s Modified Eagle Medium
AA: Arachidonic acid
PC: Phosphatidylcholine
PE: Phosphatidylethanolamine
PS: Phosphatidylserine
PI: Phosphatidylinositol
CXCL1: C-X-C motif chemokine ligand 1
