# Supplementary material for: Tumor cell death by ferroptosis contributes to an immunosuppressive tumor microenvironment in syngeneic murine models of cancer
Source: Cancer Metab. 2026 Apr 4;14:9. doi: 10.1186/s40170-026-00428-3 (PMC13072669; doi:10.1186/s40170-026-00428-3)
Supplement: Supplementary file 2 — Supplementary Material 2 [file 40170_2026_428_MOESM2_ESM.docx]

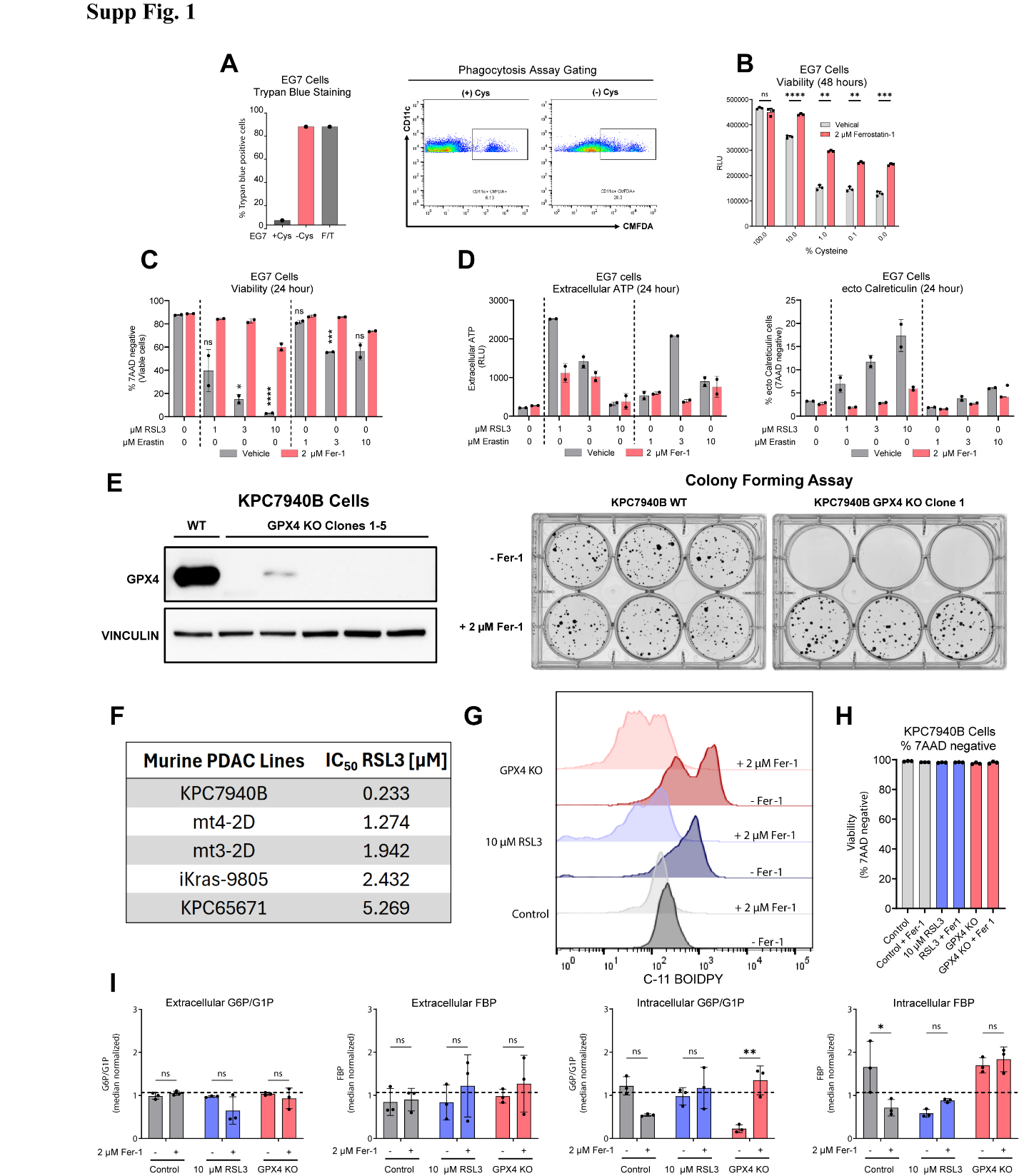


**Supplemental Figure 1. Analyses of cystine-deprivation and RSL3-treatment in murine cancer cell lines. A**, (left) Trypan Blue staining of the EG7 cells used in phagocytosis assay. EG7 cells were incubated for 48 hours in complete RPMI (+Cys), RPMI without cystine (-Cys), or subjected to freezing-thawing (F/T). (right) Flow cytometry gating of CD11^+^ CMFDA^+^ cells in phagocytosis assay. **B**, Cell viability measured using CellTiter-Glo Luminescent Cell Viability Assay, presented in relative light units (RLU), of EG7 cells grown under varied concentrations of cystine for 48 hours with or without 2 µM Fer-1, where [cystine] at 100% = 0.0652 g/L (comparable to complete RPMI). **C**, Cell viability measured by 7-AAD of EG7 cells after 24-hour treatment with 1, 3, or 10 μM RSL3 or Erastin, each dose with and without 2 μM Fer-1. **D**, (left) Extracellular ATP measured by luminescence in supernatants of EG7 cells and (right) ecto-calreticulin of 7-AAD negative EG7 cells after 24-hour treatment with 1, 3, or 10 μM RSL3 or Erastin, each dose with or without 2 μM Fer-1. **E**, (left) Western blot of KPC7940B GPK4 KO single cell clones. (right) Six-day colony forming assay of KPC7940B WT and KPC7940B GPX4 KO clone 1 cultured with and without 2 μM Fer-1. **F**, RSL3 IC_50_ concentrations in a panel of murine PDAC cell lines. **G**, Lipid peroxidation measured by C-11 BODIPY in KPC7940B after 12-hour treatment with 10 µM RSL3 or 12-hour withdrawal of 2 µM Fer-1 from GPX4-deleted KPC7940B cells. **H**, Viability of KPC7940B cells used in metabolomics as measured by 7-AAD. **I**, Mass spectrometry analysis of glucose-phosphate or fructose 1,6-bisphosphate detected in KPC7940B cells treated with or without 10 μM RSL3 or GPX4 KO cells cultured with or without 2 μM Fer-1 for 4 hours. Individual data points are presented over bar graphs with error bars, which represent the mean ± SD of technical replicates, where ns is not significant, *P* ≥ 0.05; **P* < 0.05; ***P* < 0.01; ****P* < 0.001; *****P* < 0.0001.


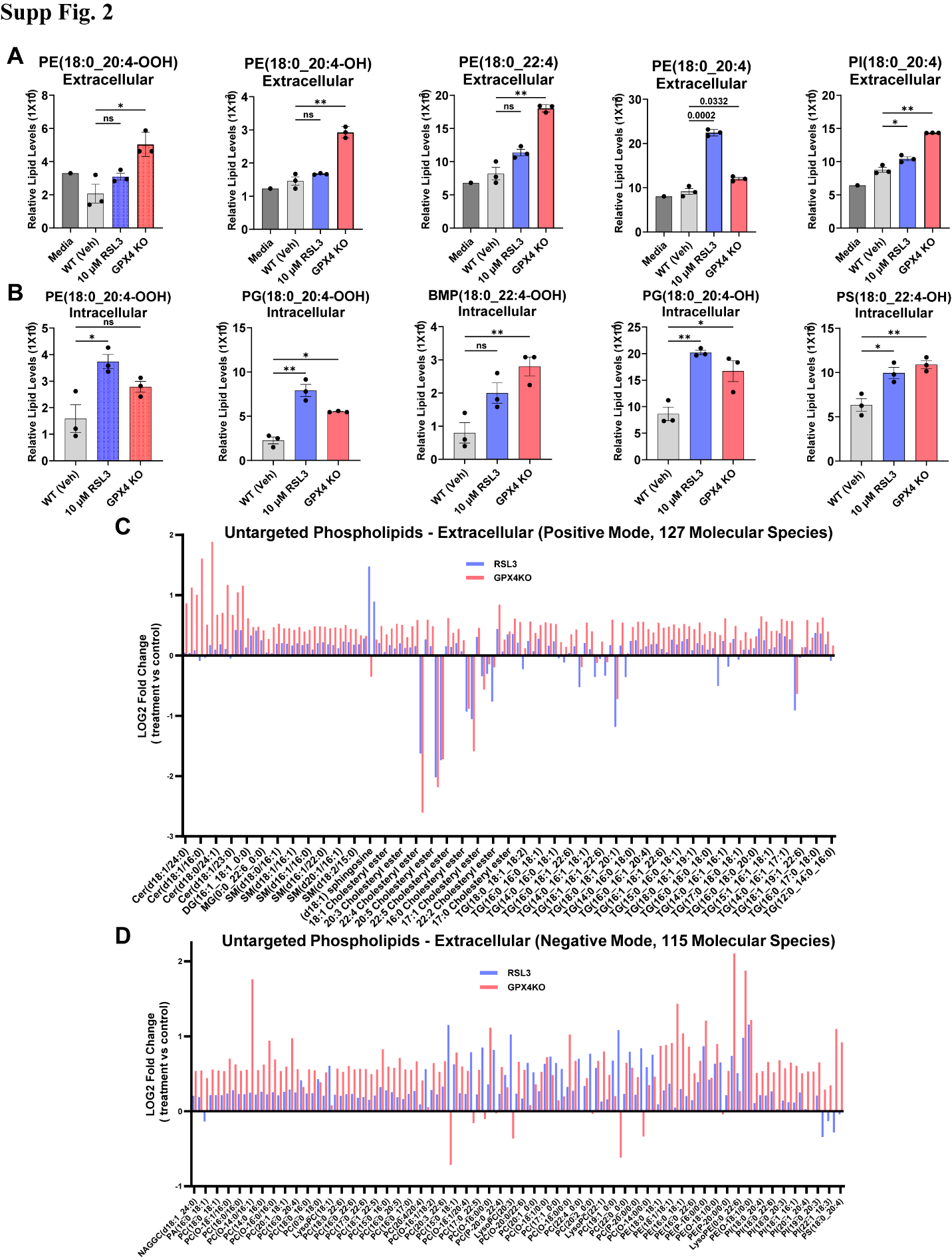


**Supplemental Figure 2. Targeted lipidomics analysis of ferroptotic PDAC cells. A**, Panel showing relative lipid levels in cell-free media, and supernatants of WT (Veh), RSL3-treated, and GPX4 KO KPC7940B cells detected by targeted mass spectrometry. **B**, Panel showing relative lipid levels in cell pellets of WT (Veh), RSL3-treated, and GPX4 KO KPC7940B cells. **C**, Log2 fold change of oxidized phospholipids detected by untargeted mass spectrometry (positive ion mode) in extracellular samples from RSL3-treated and GPX4 KO KPC7940B cells as compared to WT (Veh) control. **D**, Log2 fold change of oxidized phospholipids detected by untargeted mass spectrometry (negative ion mode) in extracellular samples of RSL3-treated and GPX4 KO KPC7940B cells as compared to WT (Veh) control. All data are presented as the mean ± SD of technical replicates, where ns, not significant, *P* ≥ 0.05; **P* < 0.05; ***P* < 0.01; ****P* < 0.001; *****P* < 0.0001.


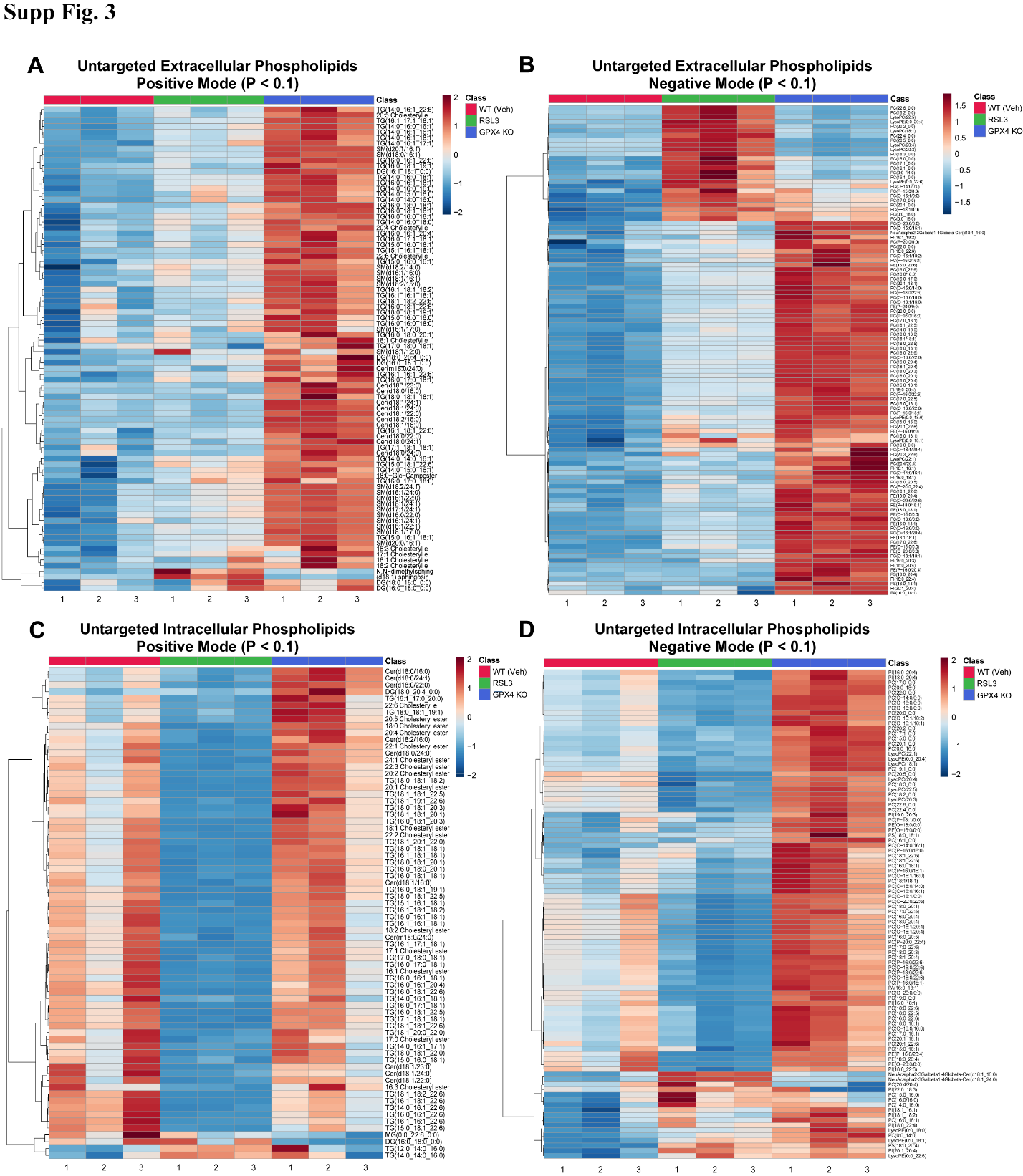


**Supplemental Figure 3. Heatmaps of lipidomics analysis in ferroptotic PDAC cells. A,B**, Heatmap showing differential (P < 0.1) oxidized phospholipids detected by untargeted mass spectrometry in positive ion mode (**A**) or in negative ion mode (**B**) in extracellular extracts of RSL3-treated and GPX4 KO KPC7940B cells as compared to WT (Veh) control. Log10 fold change is shown. **C, D,** Heatmap showing differential (P < 0.1) oxidized phospholipids detected by untargeted mass spectrometry in positive ion mode (**C**) or in negative ion mode (**D**) in cell pellets of RSL3-treated and GPX4 KO KPC7940B cells as compared to WT (Veh) control. Log10 fold change is shown.


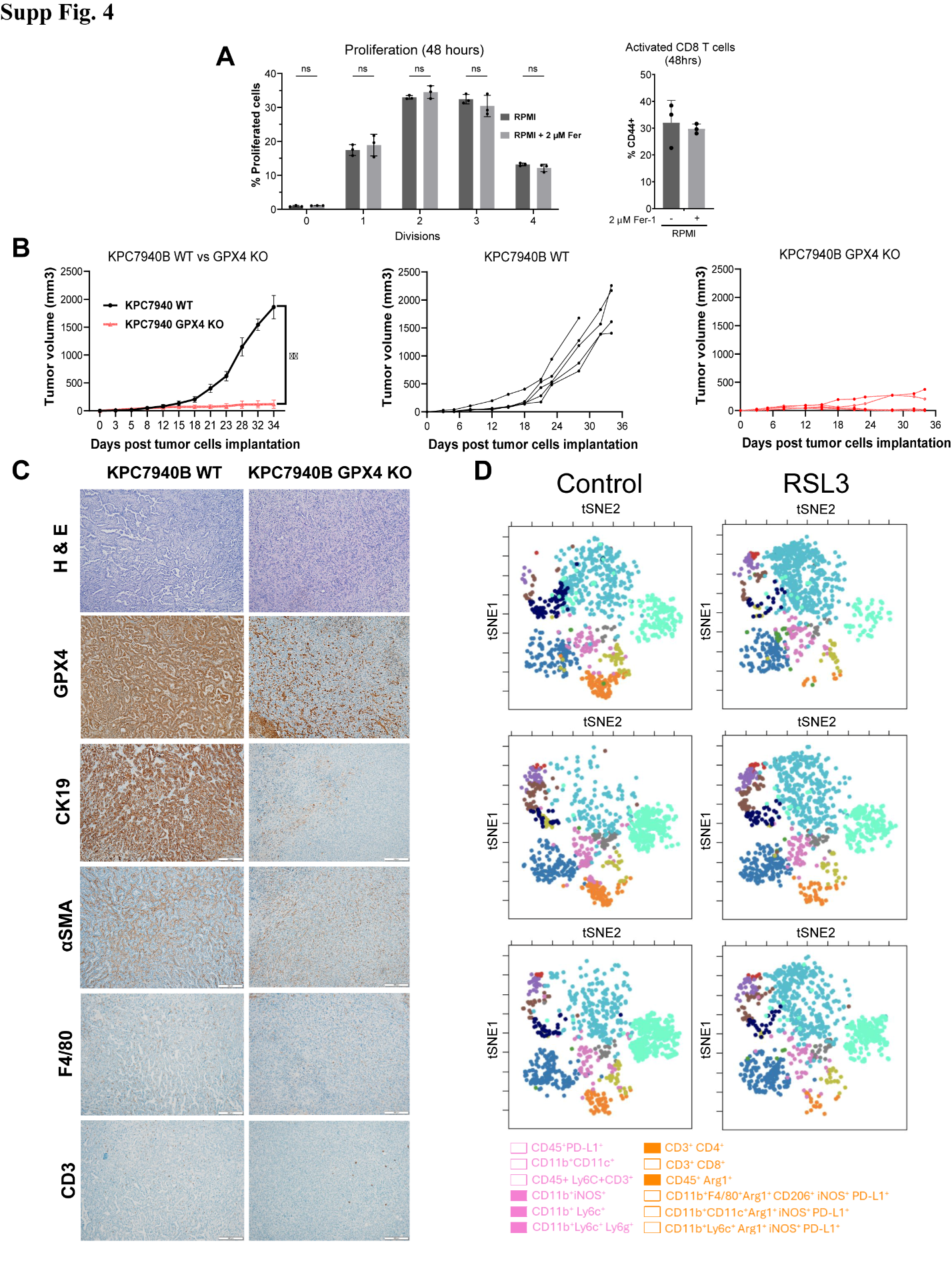


**Supplemental Figure 4. T cell and tumor analyses for the effects of PDAC ferroptotis. A**, (left) Proliferation of CD8^+^ T cells cultured in complete RPMI with or without 2 μM Fer-1, measured by flow cytometry using Cell Tracker. (right) CD44^+^ CD8^+^ T cells were measured for these conditions. Data are presented as the mean ± SD of technical replicates. **B**, Subcutaneous tumor volume in KPC7940B WT and KPC7940B GPX4 KO tumors, plotted as groups (left) and by individual mice (middle, right). Data are presented as the mean ± SD of biologically independent samples. **C**, Representative immunohistology staining of KPC7940B WT and GPX4 KO tumors from (**B**). **D**, CyTOF analysis of three subcutaneous tumors from three separate mice in the RSL3-killed group and control group from Fig. 5C. Mice in the mitoxantrone condition did not develop tumors; therefore, CyTOF analysis could not be completed for this treatment condition. ns, not significant, *P* ≥ 0.05; **P* < 0.05; ***P* < 0.01; ****P* < 0.001; *****P* < 0.0001.

**Supplementary File 1**. Raw metabolomics data used for the presentation of data in Fig. 3 and Supplementary Fig. 1I.

**Supplementary File 2**. Raw targeted lipidomics data used for the presentation of data in Fig. 3 and Supplementary Fig. 2.

**Supplementary File 3**. Raw untargeted lipidomics data used for the presentation of data in Fig. 3 and Supplementary Fig. 2.

**Supplementary File 4**. Unprocessed western blot images corresponding to the data from Supplementary Figure 1E.
