# Supplementary material for: Tumor cell death by ferroptosis contributes to an immunosuppressive tumor microenvironment in syngeneic murine models of cancer
Source: Cancer Metab. 2026 Apr 4;14:9. doi: 10.1186/s40170-026-00428-3 (PMC13072669; doi:10.1186/s40170-026-00428-3)
Supplement: Supplementary file 5 — Supplementary Material 5 [file 40170_2026_428_MOESM5_ESM.pptx]

## Slide 1
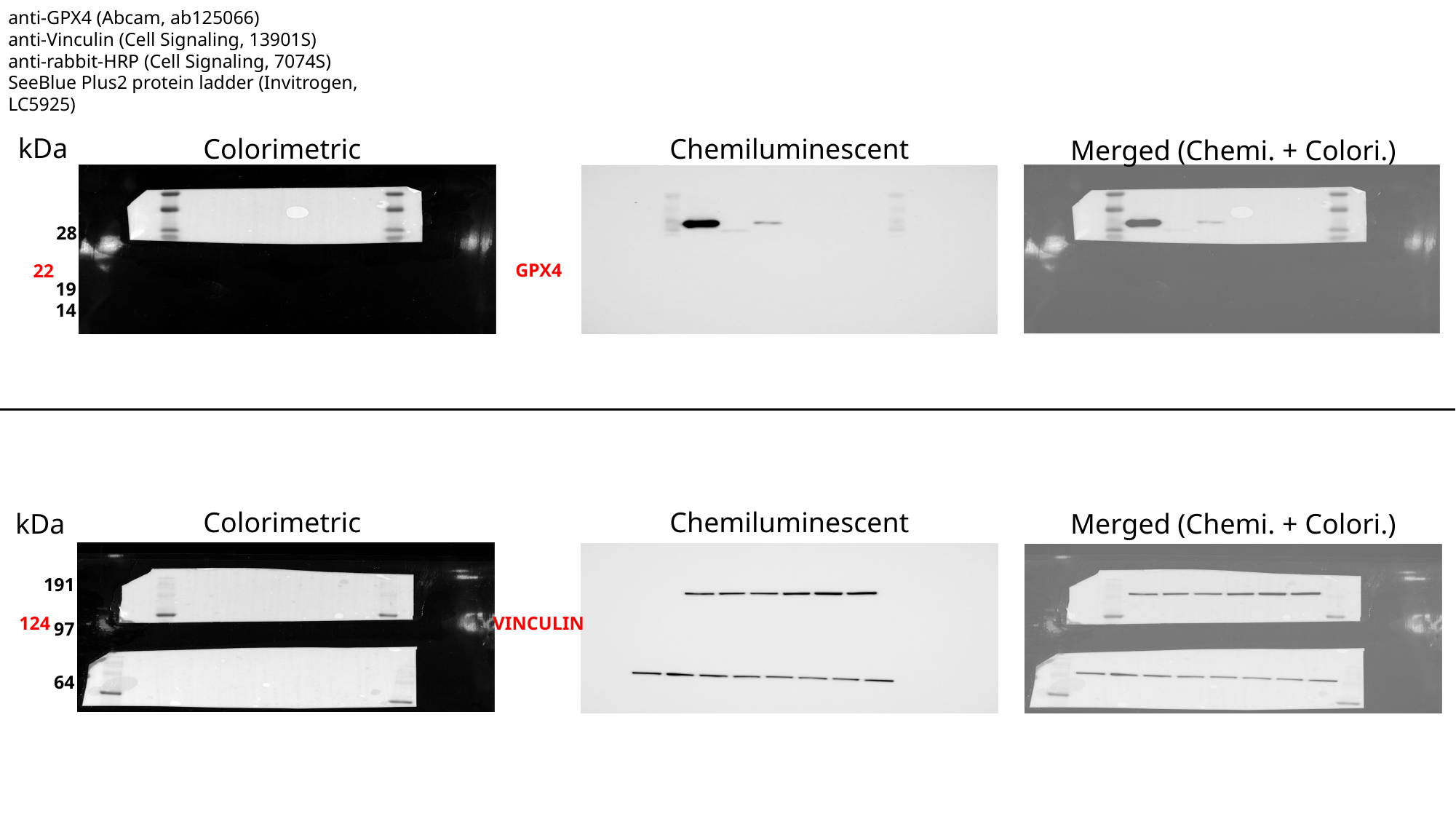

anti-GPX4 (Abcam, ab125066)
anti-Vinculin (Cell Signaling, 13901S)
anti-rabbit-HRP (Cell Signaling, 7074S)
SeeBlue Plus2 protein ladder (Invitrogen, LC5925)
kDa
Colorimetric
Chemiluminescent
Merged (Chemi. + Colori.)
28
GPX4
22
19
14
Colorimetric
Chemiluminescent
kDa
Merged (Chemi. + Colori.)
191
124
VINCULIN
97
64
